# Improvements in High-Density Lipoprotein Quantity and Quality Contribute to the Cardiovascular Benefits by Anti-tumor Necrosis Factor Therapies in Rheumatoid Arthritis: A Systemic Review and Meta-Analysis

**DOI:** 10.3389/fcvm.2021.765749

**Published:** 2021-10-29

**Authors:** Yonghong Luo, Xiaolei Ren, Shuwei Weng, Chunhui Yan, Qiaoxia Mao, Daoquan Peng

**Affiliations:** ^1^Department of Cardiovascular Medicine, The Second Xiangya Hospital, Central South University, Changsha, China; ^2^Department of Orthopedics, The Second Xiangya Hospital, Central South University, Changsha, China; ^3^Hunan Key Laboratory of Tumor Models and Individualized Medicine, The Second Xiangya Hospital, Central South University, Changsha, China; ^4^Department of Cardiovascular Medicine, Brain Hospital of Hunan Province, Changsha, China; ^5^Department of Cardiovascular Medicine, Loudi Central Hospital, Loudi, China

**Keywords:** tumor necrosis factor, rheumatoid arthritis, high-density lipoprotein, lipid, meta-analysis

## Abstract

**Objective:** Inflammation plays important role in atherosclerotic cardiovascular diseases (CVDs), but the interaction between the inflammation and lipid profile is largely unrevealed in humans. Patients with rheumatoid arthritis (RA) suffer from a higher risk of CVDs. Decreased total cholesterol (TC) and high-density lipoprotein (HDL) were prevalent in patients with RA. Anti-tumor necrosis factor (TNF) therapies relieve disease activity and decrease CVDs risk in RA, but their comprehensive effects on the lipid profile are unclear. This study aims to investigate the changes in blood lipid profile along time in the patients with RA accepting anti-TNF therapies by meta-analysis.

**Methods:** The MEDLINE, the Embase, and the Cochrane Central Register of Controlled Trials (CENTRAL) were searched for eligible literature. Data of lipids were classified into short-, mid-, and long-term according to treatment duration. Meta-analyses were performed to compare the lipid levels before and after treatments.

**Results:** A total of 44 records and 3,935 patients were included in the meta-analyses. Anti-TNF therapies were associated with significant increase in TC [mean difference (MD): +0.14, +0.23, and +0.26 mmol/l, respectively] and HDL (MD): +0.11, +0.12, and +0.11 mmol/l, respectively) in the short-, mid-, and long-term; anti-TNF therapies were associated with increased low-density lipoprotein (LDL) (MD: +0.06 mmol/l) and apolipoprotein A1 (ApoA1) (MD: +0.07 g/l) in the short-term, but not in the mid-term and long-term; triglyceride (TG) and apolipoprotein B (ApoB) do not change significantly in all the periods; proatherosclerotic indexes (TC/HDL, ApoB/ApoA1, and LDL/HDL) tend to decrease in the short- and mid-term, but return to baseline in the long-term after TNF inhibition.

**Conclusion:** Anti-TNF therapies were related to a long-term raised HDL level, which, together with evidence of improved HDL function, may contribute partially to the decreased CVDs risk by TNF inhibition.

## Introduction

Inflammation plays important role in atherosclerotic cardiovascular diseases (CVDs). Recently, two anti-inflammatory therapies, i.e., the interleukin-1β (IL-1β) monoclonal antibody, canakinumab and colchicine, have brought encouraging results in reducing the residual risks in atherosclerosis in addition to and independent of lipid-lowering effect (1–3). However, the interaction between the inflammation and lipid profile is largely unrevealed in humans.

Patients with rheumatoid arthritis (RA) are at higher risk of CVDs, which can be partially explained by traditional risk factors of CVDs including smoking, hypertension, obesity, and diabetes. Interestingly, lipid profile in the patients with RA changes toward an “atheroprotective” direction. Patients with RA have lower blood total cholesterol (TC), low-density lipoprotein (LDL), and high-density lipoprotein (HDL) compared to the general population. The lower TC and LDL are unexpectedly associated with higher CVD risk, which is called the lipid paradox (4). Some of the anti-RA therapies are associated with altered lipid profile and decreased CVDs risk, indicating that chronic systemic inflammation contributes to the lipid paradox and additional risk of CVDs (5–7). Among the anti-RA therapies, inhibition of tumor necrosis factor (TNF), an important proinflammatory cytokine that contributes to RA development, effectively ameliorates RA activity and reduces CVDs risk in the patients with RA (8). The mechanisms behind the CVD-protective role of anti-TNF therapies are poorly understood. Previous meta-analyses imply that an increase in HDL after anti-TNF therapies may contribute to CVD amelioration. However, the concomitant increase in TC, which is atherogenic, contradicts the potential benefits of HDL (9–11). Besides, LDL and triglyceride (TG) change vary among studies, making it confusing to interpret the effects of changes in the comprehensive lipid profile on CVDs. Lipid ratios, including atherogenic index (AI) (TC/HDL), apolipoprotein B/apolipoprotein A1 (ApoB/ApoA1), and LDL/HDL, may help to determine a combined effect of the changes in both the anti- and proatherogenic lipids, but previous meta-analyses seldom analyzed them systematically because of limited data. In addition, lipid changes after long-term anti-TNF treatments are seldom evaluated.

This study aims to systematically assess the changes in blood lipid profile after short-, mid-, and long-term anti-TNF therapies in patients with RA by meta-analysis of the literature. We also aim to review the evidence of altered lipoprotein functions after TNF inhibition in patients with RA, trying to investigate the possible effects of systemic inflammation inhibition on lipid profile, especially HDL.

## Methods

### Search Strategy

Refer to Supplementary Material 1.

### Study Selection

Study selection was performed by YL and XR independently. When divergency comes, a third person DP would make the final decision. Studies meet all the following criteria that were included for meta-analysis: (1) prospective studies; (2) with patients diagnosed with RA; (3) including any one of the anti-TNF therapies: infliximab (IFX), etanercept (ETN), adalimumab (ADA), certolizumab, or golimumab; and (4) with mean, SD, sample size, and treatment duration of any one of the following lipid profile both before and after anti-TNF therapies are available (either directly available, can be estimated as described in the “data extraction” section, or available from the authors): TC, TG, HDL, LDL, TC/HDL (AI), ApoA1, ApoB, ApoB/ApoA1 ratio, or LDL/HDL ratio.

### Quality Assessment of Studies

Quality assessment was performed by SW and CY independently. When divergency comes, a third person DP would make the final decision. Quality assessment was a combination based on: (1) the Cochrane Collaboration's tool for assessing the risk of bias in randomized trials (12), (2) the Newcastle–Ottawa Scale (NOS) for the cohort studies (13), (3) the methodological index for nonrandomized studies (MINORS) (14), (4) the quality assessment of diagnostic accuracy studies (QUADAS) tool for the diagnostic studies (15), and (5) potential factors that may affect lipid profile in the patients with RA. A total of 14 entries are evaluated for each included study as shown in Supplementary Table 3. Studies with at least nine scores are considered as high quality.

### Data Extraction

Data extraction was performed by YL and XR independently. When inconsistency arose, a third person QM would double check the original reference. Mean ± SD of lipid profile in the patients with RA before (baseline) and after (endpoint) anti-TNF therapies was extracted. When mean ± SD of lipid profile was not available in the articles or from the authors, mean was estimated from median if available (16) and SD was estimated from either SEM (17), interquartile range (IQR) (16), range (16), or CI (17). When either SD of baseline or endpoint was missing, estimate one from the other. When only baseline and change from baseline data were available, endpoint data were estimated by using baseline and change from baseline data (17). TC, HDL, and LDL levels in mg/ml were converted to mmol/l by multiplying by 0.02586; TG level in mg/ml was converted to mmol/l by multiplying by 0.01129. The effects of anti-TNF therapies on lipid profile were divided into short-term (0–12 weeks), mid-term (13–26 weeks), and long-term (>26 weeks) effects. For reports that present lipid data from the same clinical trial, use the data from the latest report. For the short-term and mid-term effects, if lipid data at multiple time points were available, choose the latest time point for analysis. For the long-term effect, if lipid data at multiple time points were available, choose the latest time point if the latest time point was less than 1 year; otherwise, choose the earliest time point (in this way, we are able to let the long-term treatment duration reside in a similar period around 1 year as close as possible).

### Statistical Analysis

Mean differences (MDs) with corresponding 95% CIs were calculated by using a fixed-effects model when low heterogeneity was indicated; otherwise, meta-analyses were performed by using a random-effects model. Heterogeneity was assessed by using the *I*^2^ statistic. Low heterogeneity was defined as *I*^2^ < 50% and *p* > 0.05 (17). In case of heterogeneity, subgroup analyses were performed according to quality score (≥9 vs. <9); study design (randomized controlled study vs. prospective cohort study); RA duration (≥6 vs. <6 months) (18); the Disease Activity Score-28 (DAS28) (>5.1 vs. ≤ 5.1) (18); age (≥55 vs. <55 years old); drug (IFX, ADA, ETN, and mixed); baseline lipid level (for TC: ≥5.2 vs. <5.2 mmol/l, for TG: ≥1.7 vs. <1.7 mmol/l, for HDL: >1.5 vs. ≤ 1.5 mmol/l, and for LDL: ≥3.4 vs. <3.4 mmol/l) (19). For lipids with more than 10 studies available for analysis (17), meta-regression was also performed to discover the potential sources of heterogeneity. Sensitivity analyses were performed to evaluate the robustness of the meta-analyses. The funnel plot, Begg's test, and Egger's test were performed to evaluate the potential publication bias when the number of studies included in the meta-analyses was no less than 10 (17). All the analyses were performed by Stata 12.0 software, Texas, USA.

## Results

### Search Results

**Figure 1:**
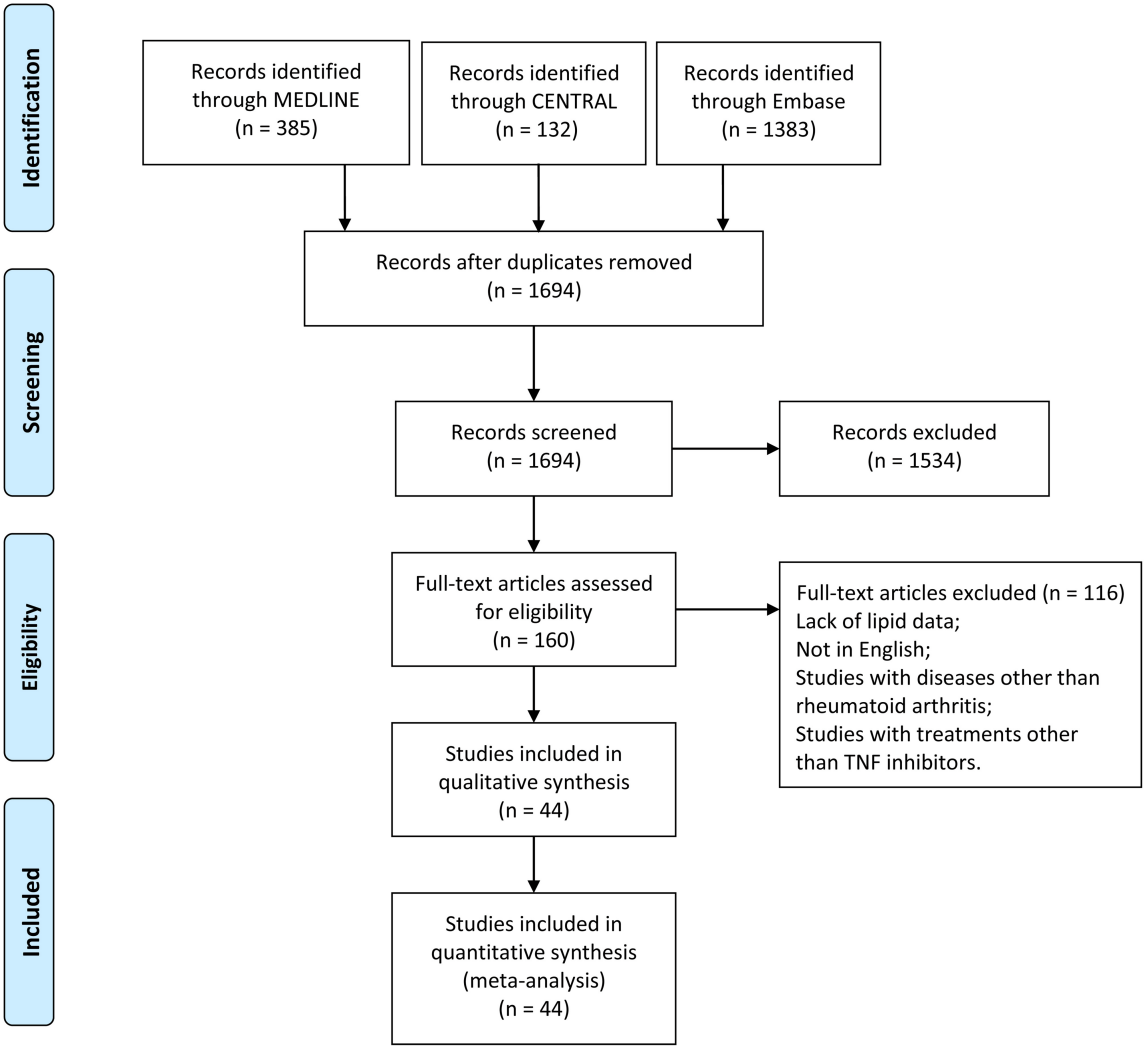
Flow diagram of studies identified, included, and excluded. CENTRAL, Cochrane Central Register of Controlled Trials.

A total of 1,900 records were obtained from the primary search of the three databases. After removing duplicates, titles and abstracts of 1,694 records were screened, among which 1,534 records were excluded according to the inclusion criteria. Full text of 160 records was read, and finally, 44 records were included in the meta-analyses (Figure 1).

### Study Characteristics and Quality Assessment

The study design, number of patients, female proportion, disease duration, the DAS28, age of the patient, drugs used, follow-up duration, lipid measurement, and lipid profile in each study were collected (Table 1). The baseline concomitant uses of drugs that may affect blood lipids were also listed including conventional disease-modifying antirheumatic drugs (cDMARDs), corticosteroids, and lipid-lowering drugs (e.g., statins) (Supplementary Table 1). A total of 3,935 patients were included in the study. Among the 44 studies included, 11 studies are randomized controlled trials (RCTs) and 33 studies are prospective cohort studies. Clinical trial registration numbers for RCTs are available in Supplementary Table 2. Six studies have more than one arm of the patients treated with anti-TNF therapies and each arm was considered an independent study. Most of the patients are female with RA duration for more than 6 months and with high disease activity (DAS28 > 5.1). Patients were treated with either IFX, ETN, or ADA and followed up for at least 2 weeks. Patients treated with golimumab or certolizumab (except in studies with mixed anti-TNF therapies) were not included in the current meta-analysis because of ineligibility or insufficient data for meta-analysis. For most of the included studies, fasting blood was used to measure lipids.

**Table 1:** Summary of the included studies.

**Study**	**Design**	**Patients**	**Female**	**RA duration^**a**^**	**DAS28^**b**^**	**Age^**c**^**	**Drug**	**Follow-up^**d**^**	**Lipid^**e**^**	**Outcomes**
Masic et al. (20)	RCT	86	61.6%	83 (43–132)^#^	5.6 ± 1.2	53.4 ± 15.7	ADA	52w	NF	TC, TG, HDL, LDL
Giles et al. (21)	RCT	1542	78.0%	7.2 (3.1–4.6)	-	61 ± 8	ETN	12, 24, 48w	NF	TC, TG, HDL, LDL
Corrado et al. (22)	cohort	10	90%	-	3.85 ± 0.9	49 ± 13.06	ADA	12, 24w	F	TC, TG, HDL, LDL, AI, LDL/HDL
Corrado et al. (22)	cohort	11	91%	-	4.05 ± 0.9	54.18 ± 10.9	IFX	12, 24w	F	TC, TG, HDL, LDL, AI, LDL/HDL
Corrado et al. (22)	cohort	12	75%	-	3.75 ± 0.4	53.42 ± 5.75	ETN	12, 24w	F	TC, TG, HDL, LDL, AI, LDL/HDL
Virone et al. (23)	RCT	96	83.3%	-	5.0 (4.1–5.6)	56.0 (45.4–64.3)	Mixed	24w	NF	Apo A1
Bergstrom et al. (24)	cohort	14	78.6%	9.0 (2.6–11.6)	5.6 ± 1.3	63.7 ± 8.9	ADA	12w	F	TC, TG, HDL, LDL, LDL/HDL, Apo B, Apo A1, Apo B/A1
Rodriguez-Carrio et al. (25)	cohort	13	92.3%	-	5.08 ± 1.93	43 (30–65)	Mixed	12w	F	TG, HDL
O'Neill et al. (26)	RCT	11	72.7%	6m- 3y	4.68 ± 1.56	61.30 ± 11.05	IFX	46w	F	TC, TG, HDL, LDL
Gabay et al. (27)	RCT	145	82%	6.3 ± 6.9	6.8 ± 0.9	53.3 ± 12.4	ADA	8w	F	TC, TG, HDL, LDL, AI
Charles-Schoeman et al. (28)	RCT	141	71.84%	3.30 ± 5.70^*^	5.82 ± 1.07	50.87 ± 12.58	ETN	24, 48, 102w	NF	TC, HDL, LDL
Charles-Schoeman et al. (28)	RCT	104					ETN	24, 78w	NF	TC, HDL, LDL
Bissell et al. (29)	RCT	35	68%	1.00 (0.72–1.45)^*^	-	52.3 ± 13.0	IFX	26, 78w	F	HDL, LDL, AI, Apo B
Deodhar et al. (30)	RCT	98	82.7%	7.4 ± 8.1	4.9 ± 0.8	55.5 ± 12.8	ETN	12w	F	TC, TG, HDL, LDL, Apo A1, Apo B
Deodhar et al. (30)	RCT	106	70.8%	8.3 ± 11.2	4.9 ± 0.7	56.5 ± 12.1	ETN	12, 24w	F	TC, TG, HDL, LDL, Apo A1, Apo B
Ronda et al. (31)	cohort	22	72.7%	9 (0.5–30)^*^	5 ± 5.8	58 ± 38.5	ADA	6, 26w	-	TC, TG, HDL, LDL
Chen et al. (32)	cohort	32	87.5%	13.4 ± 6.6	5.46 ± 0.94	53.5 ± 12.6	ADA	24w	F	TC, TG, HDL, LDL, AI
Chen et al. (32)	cohort	16	81.3%	13.6 ± 9.0	5.48 ± 0.98	54.4 ± 7.8	ETN	24w	F	TC, TG, HDL, LDL, AI
Rodriguez-Jimenez et al. (33)	cohort	22	90.9%	12.3 ± 6.7	6.2 ± 0.8	47.4 ± 8.3	ETN	4, 24w	-	TC, TG, HDL, LDL
Cacciapaglia et al. (34)	cohort	80	81.3%	7 ± 5	4.7 ± 1.6	53 ± 13	Mixed	24w, 52w	-	TC, TG, HDL, LDL, AI
Calvo Alen et al. (35)	cohort	19	68%	10.4 ± 24.7	5.5 ± 1.2	60.7 ± 13.2	Mixed	26w	-	TC
Hjeltnes et al. (36)	cohort	30	73%	8 ± 8	-	58 ± 8	Mixed	6w, 26w	F	TC, TG, HDL, LDL, Apo A1
Chen et al. (37)	cohort	20	90%	6.6 ± 5.6	>5.1	53.8 ± 11.8	ETN	12, 52w	F	TC, TG, HDL, LDL
Sandoo et al. (38)	cohort	23	65%	11 ± 11	-	54 ± 15	Mixed	12w	F	TC, TG, HDL
Sene et al. (39)	cohort	16	81.3%	9 (2–39)	6.31 (5.28–7.94)	48 (27–69)	ETN	26w	F	TC, TG, HDL, LDL
Tam et al. (40)	RCT	20	95%	4.2 (3.1–8.6)^*^	5.1 ± 0.7	53 (47–61)	IFX	26w	F	TC, TG, HDL, LDL, AI
Ajeganova et al. (41)	cohort	60	72.2%	7 (4–14)	5.7 ± 1.0	56.2 ± 12.4	ETN	12, 26, 52w	–	Apo B, Apo A1, Apo B/A1
Ajeganova et al. (41)	cohort	60					IFX			
Ajeganova et al. (41)	cohort	42					ADA			
Kume et al. (42)	RCT	21	85.7%	11 ± 5^*^	5.17 ± 1.5	61 ± 15	ETN	24w	F	TC
Kume et al. (42)	RCT	21	85.7%	9 ± 5^*^	5.34 ± 1.4	63 ± 17	ADA	24w	F	TC
Jamnitski et al. (43)	cohort	266	82%	8 (3–16)	5.21 ± 1.32	52.8 ± 12.7	ETN	16, 52w	NF	TC, TG, HDL, LDL, AI, Apo B, Apo A1, Apo B/A1
Engvall et al. (44)	RCT	18	72%	4.9 ± 3.4^*^	4.8 (3.7–5.1)	56.0 (42.0–73.0)	IFX	91w	-	Apo A1, Apo B, Apo B/A1
Derdemezis et al. (45)	cohort	30	100%	12.2 ± 6.7	4.9 ± 1.3	51.8 ± 14.4	IFX	26w	F	TC, TG, HDL, LDL
Wijbrandts et al. (46)	cohort	50	76%	4.9 (2.8–12.1)	5.6 ± 1.1	51 ± 13	ADA	16w, 52w	F	TC, TG, HDL, LDL, AI, Apo B/A1
Popa et al. (47)	cohort	45	70%	7.9 ± 6.0	5.26 ± 1.24	56 ± 11	IFX	2, 26w	F	TC, TG, HDL, LDL, AI, LDL/HDL, Apo A1
Soubrier et al. (48)	cohort	29	89.7%	-	5.19 ± 0.90	57.4 ± 10.6	Mixed	14w	F	TC, TG, HDL, LDL, AI, Apo B, Apo A1, Apo B/A1
Nishida et al. (49)	cohort	97	86.6%	8.5 ± 1.5	mean: 5.4	54.2 ± 12.6	IFX	52w	-	TC, HDL
Bosello et al. (50)	cohort	10	90%	12.80 ± 9.04	6.66 ± 1.00	53.10 ± 7.79	IFX	6, 14w	F	TC, TG, HDL
Tam et al. (51)	cohort	19	100%	11 ± 7	5.31 ± 1.06	49 ± 10	IFX	6, 14w	F	TC, TG, HDL, LDL, AI, Apo B, LDL/HDL
Popa et al. (52)	cohort	55	72.7%	9 ± 7	5.26 ± 1.25	56 ± 11	IFX	2, 26, 52w	F	TC, TG, HDL, LDL, AI, LDL/HDL, Apo B, Apo A1
Peters et al. (53)	cohort	80	77.5%	10 (0–59)	5.7	56 ± 14	IFX	6, 22, 48w	NF	TC, TG, HDL, AI, Apo A1, Apo B, Apo B/A1
Oguz et al. (54)	cohort	7	85.7%	6.8	5.8 ± 0.9	44.6 ± 12.3	IFX	mean: 42w	F	TG, HDL, TG/HDL
Komai et al. (55)	cohort	15	86.7%	10.0 ± 2.3	5.07 ± 0.77	50 ± 3	IFX	6, 26, 52w	-	TC, TG, HDL, LDL
Seriolo et al. (56)	cohort	34	100%	14 ± 9	6.9 ± 2.1	51.6 ± 7.9	Mixed	16w, 24w	F	TC, TG, HDL, AI
Dahlqvist et al. (57)	cohort	52	78.8%	14.1 ± 8.6	5.9 ± 0.72	54.6 ± 12.5	IFX	12, 26, 52w	-	TC, HDL, AI, LDL/HDL
Allanore et al. (58)	cohort	56	91%	13 ± 7	-	52 ± 14	IFX	6w, 30w	F	TC, TG, HDL, LDL, AI, LDL/HDL
Gonzalez-Juanatey et al. (59)	cohort	8	87.5%	20 (7–29)	5.5 ± 1.3	51 (24–74)	ADA	12w	F	TC, HDL, AI
Spanakis et al. (60)	cohort	24	70.8%	14.1 ± 7.2^*^	6.9 ± 1.3	62.7 ± 10.1	IFX	12, 26w	F	TC, HDL
Popa et al. (61)	cohort	33	-	-	5.24 ± 1.05	-	ADA	2w	F	TC, TG, HDL, LDL, AI, LDL/HDL
Vis et al. (62)	cohort	69	80%	12 (0–59)	5.9 ± 1.4	58 (24–80)	IFX	6w	NF	TC, HDL, AI
Irace et al. (63)	cohort	10	60%	7 ± 2	3.4 ± 1.3	46 ± 12	IFX	6w	F	TC, TG, HDL, Apo A1, Apo B

a *Data are mean ± SD, median [interquartile range (IQR)], range, median (range), or mean (range) in years, unless otherwise specified*;

* *in months*;

# *in days*.

b *Data are mean ± SD, mean, median, median (IQR), or median (range), unless otherwise specified; if both Disease Activity Score-28-C-reactive protein (DAS28-CRP) and DAS-28-erythrocyte sedimentation rate (DAS28-ESR) are provided, DAS28-ESR is presented*.

c *Data are mean ± SD, median (IQR), or median (range) in years unless otherwise specified*.

d *When follow-up duration is not presented as weeks, convert it to weeks by using: 1 month = 4 weeks, 3 months = 12 weeks, 6 months = 26 weeks, and 1 year = 52 weeks; only time points that are included in the meta-analysis are presented*.

e *F = lipids measured by using the fasting blood samples; NF = lipids measured by using the non-fasting blood samples*.

The quality of the included studies was assessed by the quality assessment lists consisted of 15 items that may affect the lipid outcomes (Supplementary Table 3). About nine studies are assessed as low quality (quality <9).

### Primary Outcomes

#### Short-Term Changes in Lipid Profile

**Figure 2:**
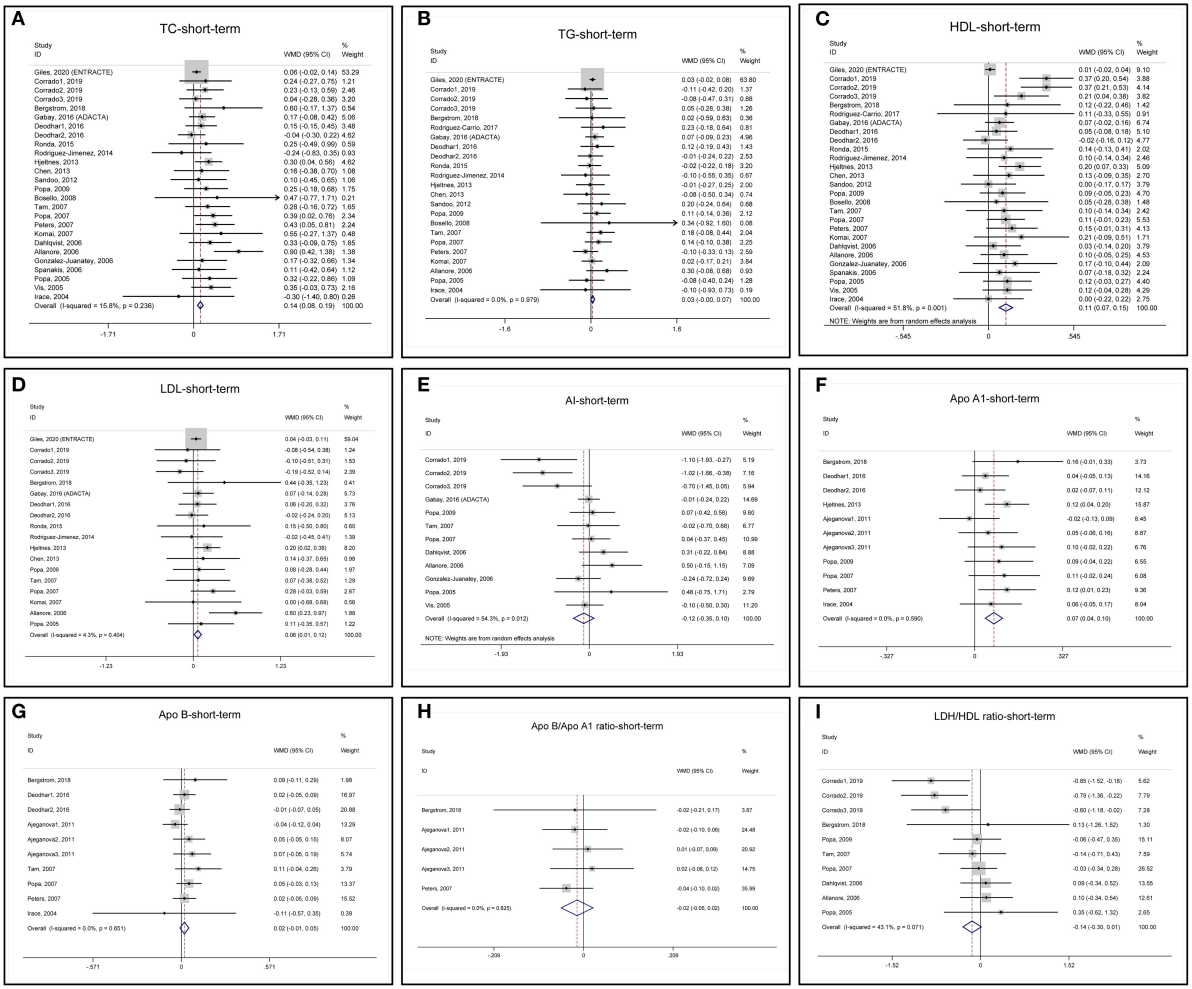
Forest plots of meta-analyses of the short-term changes in **(A)** total cholesterol (TC), **(B)** triglyceride (TG), **(C)** high-density lipoprotein (HDL), **(D)** low-density lipoprotein (LDL), **(E)** atherogenic index (AI), **(F)** ApoA1, **(G)** ApoB, **(H)** ApoB/ApoA1 ratio, and **(I)** LDL/HDL ratio.

Short-term changes in lipid profile after anti-TNF treatments are summarized in Table 2 and the forest plots are shown in Figure 2. Short-term anti-TNF treatments are associated with a significant increase in blood TC (Figure 2A), LDL (Figure 2D), and ApoA1 (Figure 2F) without heterogeneity. Blood TG (Figure 2B) and ApoB (Figure 2G) show no significant changes without heterogeneity. HDL increased significantly with heterogeneity (Figure 2C). AI does not change with heterogeneity (Figure 2E). ApoB/ApoA1 ratio (Figure 2H) and LDL/HDL ratio (Figure 2I) tend to decrease but do not reach statistical significance without heterogeneity.

**Table 2:** Summary of the meta-analyses of changes in lipid profile.

	**Number of study**	**Number of patients**	**WMD (95% CI)**	**Heterogeneity *I*^**2**^, *p*-value**
**Short-term**				
TC (mmol/l)	26	2530	0.14 (0.08, 0.19)	15.8%, 0.236
TG (mmol/l)	23	2390	0.03 (−0.00, 0.07)	0.0%, 0.979
HDL (mmol/l)	27	2542	0.11 (0.07, 0.15)	51.8%, 0.001
LDL (mmol/l)	18	2253	0.06 (0.01, 0.12)	4.3%, 0.404
AI	12	515	−0.12 (−0.35, 0.10)	54.3%, 0.012
Apo A1 (g/l)	11	600	0.07 (0.04, 0.10)	0.0%, 0.590
Apo B (g/l)	10	544	0.02 (−0.01, 0.05)	0.0%, 0.651
Apo B/Apo A1	5	256	−0.02 (−0.05, 0.02)	0.0%, 0.825
LDL/HDL	10	307	−0.14 (−0.30, 0.01)	43.1%, 0.071
**mid-term**				
TC (mmol/l)	30	2918	0.23 (0.10, 0.36)	73.1%, 0.000
TG (mmol/l)	23	2536	0.01 (−0.03, 0.04)	14.0%, 0.270
HDL (mmol/l)	29	2906	0.12 (0.06, 0.19)	84.0%, 0.000
LDL (mmol/l)	23	2692	0.06 (−0.04, 0.15)	52.1%, 0.002
AI	16	766	−0.12 (−0.29, 0.06)	64.4%, 0.000
Apo A1 (g/l)	11	869	0.03 (−0.00, 0.06)	0.0%, 0.981
Apo B (g/l)	10	752	0.00 (−0.02, 0.02)	0.0%, 0.720
Apo B/Apo A1	7	587	−0.02 (−0.05, 0.00)	0.0%, 0.940
LDL/HDL	7	204	−0.28 (−0.67, 0.10)	78.4%, 0.000
**long-term**				
TC (mmol/l)	14	2639	0.26 (0.02, 0.49)	86.9%, 0.000
TG (mmol/l)	11	2252	0.03 (−0.05, 0.11)	47.0%, 0.042
HDL (mmol/l)	17	2695	0.11 (0.04, 0.19)	85.4%, 0.000
LDL (mmol/l)	12	2445	0.10 (−0.05, 0.24)	69.2%, 0.000
AI	7	594	0.03 (−0.11, 0.17)	19.1%, 0.284
Apo A1 (g/l)	7	581	0.03 (−0.01, 0.07)	25.5%, 0.234
Apo B (g/l)	8	616	0.01 (−0.02, 0.03)	0.0%, 0.877
Apo B/Apo A1	7	576	−0.02 (−0.04, 0.01)	0.0%, 0.789
LDL/HDL	3	163	0.11 (−0.12, 0.34)	0.0%, 0.430

To test the robustness of the meta-analyses, we performed the sensitivity analyses (Supplementary Figure 1). The ENTRACTE trial greatly influences the variation of pooled MD of TC, TG, HDL, and LDL because of a large patient number, but it does not change the direction of the results (Supplementary Figures 1A–D). After removing the ENTRACTE trial, the pooled MD of TC, HDL, and LDL increases more. The results of AI, ApoA1, ApoB, ApoB/ApoA1 ratio, and LDL/HDL ratio are not affected by removing any one of the included studies (Supplementary Figures 1E–I).

The funnel plot, Begg's test, and Egger's test show that the pooled MD of TC and HDL is at high risk of publication bias, while another lipid profile is at low risk of publication bias (Supplementary Figures 2A–I). Because the ENTRACTE trial greatly influences the robustness of TC and HDL meta-analyses (Supplementary Figures 1A,C), we removed the ENTRACTE study and reperformed the analyses. Removing the ENTRACTE trial results in the symmetrical funnel plot and nonsignificant *p*-values of the Begg's test and Egger's test for TC and HDL (Supplementary Figure 3). Besides, removing the ENTRACTE study also significantly decreases the heterogeneity of the meta-analysis of short-term HDL changes (Supplementary Figure 3C), indicating the ENTRACTE trial as a potential source of heterogeneity. We re-examined the characteristics of the ENTRACTE trial. We found that the trial included patients with at least one traditional CVD risk factor or a history of CVD events, which may bring confounding factors that affect lipid profile (21). Also, concomitant use of cDMARDs, corticosteroids, and lipid-lowering therapies that may influence lipid profile was not well controlled in the trial, resulting in a low-quality score (Supplementary Table 3).

#### Mid-term Changes in Lipid Profile

**Figure 3:**
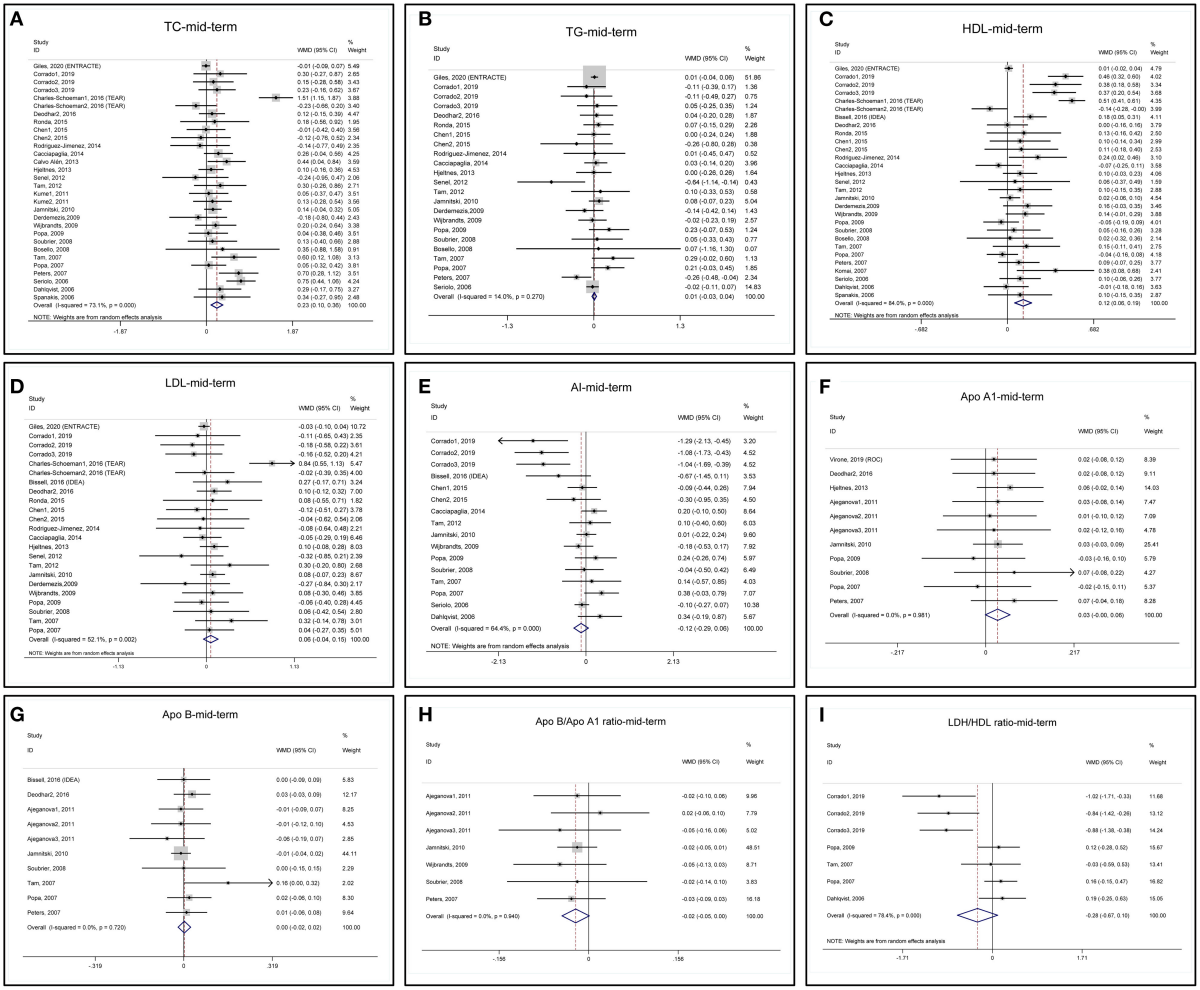
Forest plots of meta-analyses of the mid-term changes in **(A)** TC, **(B)** TG, **(C)** HDL, **(D)** LDL, **(E)** AI, **(F)** ApoA1, **(G)** ApoB, **(H)** ApoB/ApoA1 ratio, and **(I)** LDL/HDL ratio.

Mid-term changes in lipid profile after anti-TNF treatments are summarized in Table 2 and the forest plots are shown in Figure 3. Mid-term anti-TNF treatments are associated with a significant increase in TC (Figure 3A) and HDL (Figure 3C) with heterogeneity. Similar to short-term, blood TG does not change without heterogeneity (Figure 3B). LDL (Figure 3D) and ApoB (Figure 3G) do not change after mid-term anti-TNF therapies with and without heterogeneity, respectively. ApoA1 (Figure 3F) tends to increase without heterogeneity. Mid-term anti-TNF therapies tend to decrease AI (Figure 3E), ApoB/ApoA1 ratio (Figure 3H), and LDL/HDL ratio (Figure 3I). One study presented the ApoA1 and ApoB data in mmol/l and was excluded from the meta-analyses (46).

Sensitivity analyses of TC and LDL indicate that one arm of the TEAR (TEAR1) study obviously influences the results of pooled MD (Supplementary Figures 4A,D) and removing the TEAR1 study from meta-analysis decreases the heterogeneity (Supplementary Figures 5A,B). When we checked the characteristics of the TEAR1 study (28, 64), we found a high dropout rate (31.1%) (Supplementary Table 3). Again, sensitivity analysis of TG reveals that the ENTRACTE trial significantly affects the variation, but not the pooled MD results (Supplementary Figure 4B). Sensitivity analyses of HDL, AI, ApoA1, ApoB, ApoB/ApoA1 ratio, and LDL/HDL ratio show that these indexes are not affected by removing any of the included studies (Supplementary Figures 4C,E–I).

The funnel plot, Begg's test, and Egger's test show that the pooled MD of mid-term changes in blood TC, TG, HDL, LDL, AI, ApoA1, and ApoB are at low risk of publication bias (Supplementary Figures 6A–G).

#### Long-Term Changes in Lipid Profile

**Figure 4:**
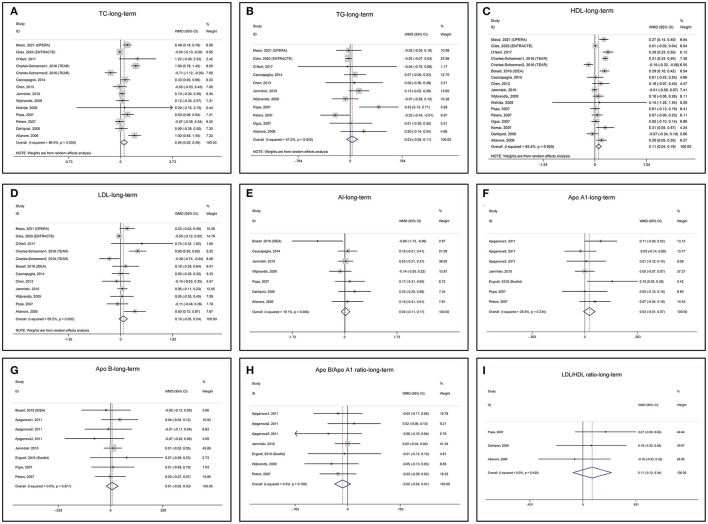
Forest plots of meta-analyses of the long-term changes in **(A)** TC, **(B)** TG, **(C)** HDL, **(D)** LDL, **(E)** AI, **(F)** ApoA1, **(G)** ApoB, **(H)** ApoB/ApoA1 ratio, and **(I)** LDL/HDL ratio.

Long-term changes in lipid profile after anti-TNF treatments are summarized in Table 2 and the forest plots are shown in Figure 4. Long-term anti-TNF treatments are associated with a significant increase in TC (Figure 4A) and HDL (Figure 4C) with heterogeneity. TG (Figure 4B) and LDL (Figure 4D) do not change after long-term anti-TNF therapies with heterogeneity. Long-term anti-TNF therapies do not change AI, ApoA1, ApoB, ApoB/ApoA1 ratio, and LDL/HDL ratio without heterogeneity (Figures 4E–I). One study presented the ApoA1 and ApoB data in mmol/l and was excluded from the meta-analyses (46).

Sensitivity analysis of TG indicates that the study by Popa (Popa-2007) (52) obviously influences the results of pooled MD (Supplementary Figure 7B) and remove it from meta-analysis decreases the heterogeneity (Supplementary Figure 8A). The heterogeneity of the Popa-2007 study may be due to a high dropout rate (43.6%) in the study (Supplementary Table 3). Again, the TEAR1 study influences the results of pooled MD for LDL (Supplementary Figure 7D) and remove it from meta-analysis decreases the heterogeneity (Supplementary Figure 8B). Sensitivity analyses of TC, HDL, AI, ApoA1, ApoB, ApoB/ApoA1 ratio, and LDL/HDL ratio show that these lipids are not affected by removing any included studies (Supplementary Figures 7A–I).

The funnel plot, Begg's test, and Egger's test show that the pooled MD of mid-term changes in blood TC, TG, HDL, and LDL are at low risk of publication bias (Supplementary Figures 9A–D).

#### Subgroup Analyses and Meta-Regression

Subgroup analyses of changes in all the lipid profiles in the short-, mid, and long-term are summarized in Supplementary Tables 4–12, where meta-regression was also presented for the lipids with more than 10 studies available for analysis.

We found that RA duration may contribute to the heterogeneity of various changes of the lipids. Subgroup analyses show that meta-analysis of lipid changes in the patients with RA duration ≥ 6 months is usually with decreased heterogeneity compared to the total population. For the patients with disease duration for more than 6 months, mid-term and long-term TNF inhibition are associated with increased HDL (MD: +0.03 mmol/l for mid-term; MD: +0.08 mmol/l for long-term) without heterogeneity (Supplementary Table 6); mid-term and long-term TNF inhibition is not associated with LDL change without heterogeneity (Supplementary Table 7); short-term and mid-term TNF inhibition is not associated with AI change without heterogeneity (Supplementary Table 8). Meta-regression also shows that RA duration may significantly contribute to the heterogeneity (*p* < 0.05 for HDL short and mid-term changes in Supplementary Table 6, for LDL mid-term change in Supplementary Table 7, and AI short and mid-term changes in Supplementary Table 8).

Subgroup analysis and meta-regression imply that IFX may be associated with a more short-term increase in TC (Supplementary Table 4). Meta-regression also indicates that baseline lipid levels may contribute to the heterogeneity for long-term TC change (Supplementary Table 4) and quality score may be a possible source of heterogeneity for long-term LDL change (Supplementary Table 7). Baseline DAS28, age of the patient, and different TNF inhibitors do not likely contribute to the heterogeneity in the current meta-analyses.

## Discussion

The current meta-analysis investigates the changes in blood lipid profile after short-term (0–12 weeks), mid-term (13–26 weeks), and long-term (>26 weeks) anti-TNF therapies in patients with RA. This study is important for some reasons. First, it adds meta-analysis of the lipid changes following long-term anti-TNF treatments based on sufficient studies, which provides evidence to evaluate the possible roles of lipid alterations in the favorable CVDs effects of TNF blockade in RA. Second, this study is a state-of-art meta-analysis that systematically analyzes apolipoproteins and several atherogenic lipid ratios including AI, ApoB/ApoA1, and LDL/HDL, which gives an overall view of the lipid changes, since previous studies have shown simultaneous elevations in both “proatherogenic” and “antiatherogenic” lipids. Third, the comprehensive subgroup analysis in this study provides insights into factors that may impact lipid profile after anti-TNF therapies, which are not provided by previous studies. Finally, we observed consistent increases in TC and HDL as the previous meta-analysis (10), but we also come to the new conclusion of TG and LDL, the changes of which varied in previous studies (65).

The mid and long-term increase in TC after TNF blockade (MD: +0.23, +0.26 mmol/l in the mid and long-term, respectively) is primarily possible due to an increase in the antiatherogenic HDL (MD: +0.12, +0.11 mmol/l in the mid and long-term, respectively), as we found no significant changes in TG, atherogenic lipids (LDL and ApoB), and atherogenic indexes (TC/HDL, ApoB/ApoA1, and LDL/HDL) after mid and long-term TNF inhibition. In general, lipid profile changes toward an atheroprotective direction. A previous meta-analysis showed a similar increase in TC and HDL in the short- and mid-term after TNF inhibition, but it did not evaluate long-term changes of the lipids (9). According to the study by Daien et al. (10), they also indicated an association between TNF inhibition and elevated HDL, elevated TC, unchanged LDL, and unchanged AI in the long-term, but it mostly included studies that using IFX, which was the prior choice of TNF inhibition at that time. We have included more studies with ADA and ETN published in recent years. Different from our results, Daien et al. found that TG level increased and ApoB/ApoA decreased after long-term inhibition, but their results were based on a very small population.

The mechanisms behind the increase of TC and HDL upon TNF inhibition are unclear. Basic researches have revealed a complex regulation between TNF and lipid metabolism, involving regulations in both hepatocytes, macrophages, and adipocytes, which need further verification in human (66). Previous evidence indicates that reduced lipid catabolism after inflammation inhibition may result in elevations in lipid levels (67).

In the current meta-analysis, to detect the possible confounding effects from the use of corticosteroids, cDMARDs, and lipid-lowering drugs (e.g., statins) that may affect the blood lipids, we included six items toward the use of these drugs in quality assessment (Supplementary Table 3), where we evaluated whether those treatments were listed at the baseline and controlled during the treatment periods. Those studies with descriptions such as “corticosteroids/MTX/statins use was stable during the study period” were considered with low risk. We also listed the baseline use of those drugs for each study in Supplementary Table 1. In this way, we try to control the confounding risk of corticosteroids/MTX/statins use within studies. However, we still cannot completely control the “interstudy” confounding risk from corticosteroids/MTX/statins use. As shown in Table 2, although MTX was the most frequently used cDMARDs, prednisolone and prednisone were the most frequently used steroids, and statins were the most frequently used lipid-lowering therapies, the dosage and the percentage of drug use among different studies varied slightly, which may cause heterogeneity among studies. In this study, we do not include unpublished data, which are usually with smaller sample sizes and uncontrolled bias due to lack of peer view (68).

We found that the meta-analyses for lipid changes in the mid and long-term are usually with heterogeneity, which may come from different RA duration as indicated by subgroup analysis and meta-regression. When checking the concomitant uses of corticosteroids, cDMARDs, and lipid-lowering drugs (Supplementary Table 1) in studies, we found that a smaller portion of the patients was treated with those drugs or treated with lower dosages in the RA duration < 6 months subgroup. This may be because that the patients with early RA suffer from less severe diseases. We also observed that the information of statin use during the TNF inhibition treatment period was usually unavailable for the studies that included the patients with early RA (see Supplementary Table 3, the “representativeness of study population” item, where the “1” superscript indicates early RA), which may be because that most of these studies were not focused on investigating the lipid changes (see the “designed to evaluate lipid levels” item). Only a small portion of studies and patients are with disease duration < 6 months possibly because that treatment-naïve patients have more choices other than TNF inhibition. Hence, it should be very cautious to interpret the results for early RA, and more studies in the future are needed.

For both HDL changes in the mid-term and long-term, we observed that meta-regression indicated marginal *p* values (*p* = 0.054 and *p* = 0.071, respectively) for baseline HDL (Supplementary Table 6). When checking baseline HDL levels for each included study, we found a very broad distribution of baseline HDL levels from 0.86 to 1.91 mmol/L (even baseline TC levels among the included studies only vary between around 4.3 and 6.4 mmol/L). We consider this huge difference (more than double) in baseline HDL levels that may account partially for the heterogeneity for changes in HDL, no matter in the short-, mid-, or long-term. Hence, caution is needed when interpreting these results.

Besides the quantitative changes in HDL after TNF inhibition, the functional changes of HDL add more complexity to the issue. By promoting cholesterol efflux, reducing inflammation, ameliorating oxidation, and improving endothelial function, HDL protects against atherosclerosis. As shown in this meta-analysis, the consistent increase in HDL in all the periods may partially account for the reduced CVDs risk in the patients with RA accepting anti-TNF treatments. However, improvements in the functions of the HDL followed by a relief of inflammation may also contribute to the CVD benefits of TNF inhibitors.

In the inflammatory state, proinflammatory cytokines such as TNF and interleukin-6 (IL-6) significantly induce the hepatic expression of apolipoprotein serum amyloid A (SAA), which replaces ApoA1 on HDL and results in SAA-rich HDL. SAA-rich HDL was eliminated from plasma more rapidly than Apo A1-HDL (69) and showed decreased ability to induce endothelial nitric oxide (NO) production, inhibit endothelial reactive oxygen species (ROS) production, decrease inflammation, and induce cholesterol efflux (70, 71). When SAA is high, HDL cholesterol (HDL-C) inversely correlates with all-cause and cardiovascular mortality (70). In RA, HDL-associated SAA was upregulated (72) and TNF inhibition was correlated with reduced HDL-associated SAA level (27), especially in those responders. The most recent study shows that a 22% increase in anti-inflammation of HDL in the endothelial cells is associated with a 23% reduction in 10-year cardiovascular event risk. The beneficial effect is independent of the HDL level or cholesterol efflux capacity (CEC) of HDL (73). Since SAA-rich HDL exhibits impaired anti-inflammation capacity, anti-TNF therapies may decrease CVDs risk by correcting the impaired anti-inflammation function of HDL in RA.

Inflammation also impairs the expression and activity of paraoxonase 1 (PON1), the major component that accounts for the antioxidative capacity of HDL. PON1 hydrolyzes a wide range of substrates including atherogenic lipid peroxides, thus exerting atheroprotective roles. The PON1 single nucleotide polymorphism (SNP) Q192R greatly influences PON1 activity. Individuals carrying the RR allele, which associates with higher PON1 activity, had lower systemic oxidative stress and CVDs risk (74). In the patients with RA, the RR genotype correlated with decreased risk of carotid plaque (75). With respect to the general population, PON1 activity was significantly decreased, while oxidative metabolites were increased in the patients with RA, especially in patients with high disease activity (76). In mouse inflammatory arthritis models, overexpression of human PON1 decreased bioactive lipid mediators and ameliorates arthritis (77). TNF and ROS interact reciprocally in a positive feedback loop, exaggerating the inflammatory and oxidative stress in inflammatory diseases. Besides, TNF decreased PON1 expression in HepG2 cells, a hepatoma cell line (78), implying a therapeutic potential to inhibit oxidative stress by TNF inhibition. Indeed, Popa et al. proved that a 6-month TNF blockade by IFX increased PON1 activity and antioxidative capacity of HDL in the patients with RA, which correlated with a decrease in erythrocyte sedimentation rate (ESR) (47). A genome-wide association scan also identified that an SNP locus that includes the PON1 gene correlates with response to anti-TNF therapies in the patients with RA, indicating a role of PON1 in disease amelioration following TNF inhibition (79).

In contrast to PON1, myeloperoxidase (MPO), a peroxidase enzyme, facilitates ROS production and oxidizes ApoA1 on HDL, leading to dysfunctional HDL and contributing to atherosclerosis. Induced by oxidative stress and inflammation, MPO promoted the development of arthritis in an experimental mouse model (80). In the patients with RA, MPO/PON1 ratio was elevated, positively correlated with DAS28, and higher in the patients with a history of CVDs compared to the patients without a history of CVDs (81). Higher plasma MPO activity and increased MPO-oxidized HDL in RA are also associated with decreased antioxidant and cholesterol efflux capacity of HDL (82, 83). All these results imply that amelioration of inflammation in RA may decrease MPO and improve the functions of HDL.

Cholesterol efflux capacity inversely correlates with CVDs risk in the general population, independent of HDL level. HDL from the patients with RA exhibited impaired CEC (83) and a higher CEC was correlated with a lower presence of carotid plaque in the patients with RA (84). Moreover, CEC was negatively related to disease activity in RA and TNF inhibition partially restored CEC (85). TNF attenuated CEC via suppressing the expression of ABCA1, the primary molecule that mediates cholesterol efflux to ApoA1 (86). Furthermore, TNF reduced the expression of lecithin–cholesterol acyltransferase (LCAT) and cholesteryl ester transfer protein (CETP) (87, 88), two enzymes in HDL that facilitate cholesterol efflux and were decreased in RA (67, 89). The above evidence illuminates the potential of TNF inhibition to restore the CEC of HDL in RA, either by directly blocking TNF signaling or by ameliorating systemic inflammation. Indeed, reduction in high-sensitivity C-reactive protein (hs-CRP) correlates with improved CEC in RA (85). In this study, we found a significant increase in ApoAI, which may also promote cholesterol efflux. However, in a small cohort of the patients with RA, 1-year treatment of IFX failed to improve CEC in HDL significantly (26). Another small cohort study also found a non-significant increase in CEC in the patients with CEC after a 6-month treatment of ADA (31). Further studies including a larger number of patients are still needed to evaluate the effect of anti-TNF therapies on CEC in HDL.

Cardiovascular disease development in RA is partially due to endothelial dysfunction caused by multiple factors including the elevated proinflammatory cytokines (such as TNF). A meta-analysis including 20 studies demonstrates that anti-TNF treatment improves endothelial function in RA (90). HDL ameliorates endothelium injury in atherosclerosis by promoting nitric oxide (NO) production, inhibiting inflammation, apoptosis, and thrombosis, and facilitating endothelial repair. The beneficial effects of anti-TNF therapies at least partially come from improved HDL function. O'Neill et al. revealed an elevated endothelial NO bioavailability and reduced superoxide production by HDL isolated from the patients with RA who received a 2-year IFX treatment compared to HDL from those receiving placebo (26). Still, more evidence is needed.

Following TNF inhibition, the increase in HDL and ApoA1 is usually more significant in those responsive to TNF blockade (24, 52, 60, 62), corresponding to the fact that those responders gain more prominent CVDs benefits (8), suggesting a role of decreased systemic inflammation following anti-TNF therapies in the observed CVDs benefits. Currently, we cannot analyze the association between treatment response and lipid changes due to insufficient data. In this meta-analysis, the increase in HDL in the long-term (+0.11 mmol/l) seems too minor to exert beneficial cardiovascular effects. However, based on four large prospective cohorts, a 0.026 mmol/l (1 mg/dl) increment in HDL correlated with a 2–3% reduction in the risk of coronary heart disease (91). Together with improved HDL functions after anti-TNF treatments (27, 47, 85), we propose that improvements in both “quantity” and “quality” of HDL contribute to the observed cardiovascular benefits of TNF inhibition in the patients with RA. However, further evidence is needed to support that improved HDL functions after TNF inhibition promotes the reduced CVDs risk in the RA population. Alterations in LDL subgroups and modifications may also help to lower CVDs risk (92).

This study encompasses some limitations. The effects of golimumab or certolizumab on lipids are not investigated in our meta-analysis due to insufficient data. We did not include lipoprotein(a) [Lp(a)] data into the meta-analysis because few studies reported Lp(a) and a mixed presentation of Lp(a) in either mmol/l or mg/dl or logarithmic or original form in the different studies. The DAS28, we collected, is a mixture of results based on either CRP or erythrocyte sedimentation rate (ESR). However, we used a threshold of 5.1 (which is generally considered for DAS28-ESR) to differentiate the patients with high disease activity. We cannot explain the heterogeneity for long-term change in TC after TNF inhibition (though meta-regression indicated a marginal *p* = 0.063 for quality score, see Supplementary Table 4). One possibility is that other confounding factors may come up during the long-term treatment (e.g., drug use for complications of RA). Thus, caution is needed when interpreting the results.

In conclusion, the current meta-analysis shows that although anti-TNF therapies were associated with a significant increase in blood TC in the long-term in RA, atherogenic lipids (LDL and ApoB) and atherogenic indexes (AI, ApoB/ApoA1, and LDL/HDL) do not increase after long-term TNF blockade. HDL mostly contributes to the increased TC. Together with evidence that inflammation impairs HDL functions and inflammation reduction is associated with improved HDL functions, it is likely that improvements in HDL quantity and quality at least partially contribute to the reduced CVDs risk by TNF inhibition. Our findings add to evidence that systemic inflammation inhibition may exert a favorable alteration in lipid profile and HDL function in the patients with a highly inflammatory state, thus potentiating to reduce the CVD risk in those patients. However, direct evidence is still required in the future.

## Data Availability Statement

The raw data supporting the conclusions of this article will be made available by the authors, without undue reservation.

## Author Contributions

YL and DP come up with the concept and design of the study. YL, XR, SW, CY, and QM performed the data collection. YL performed the statistical analysis, did the interpretation, and drafted the manuscript. DP revised the manuscript. All the authors contributed to the article and approved the submitted version.

## Funding

This study was supported by grants from the National Natural Science Foundation of China (No. 81870336).

## Conflict of Interest

The authors declare that the research was conducted in the absence of any commercial or financial relationships that could be construed as a potential conflict of interest.

## Publisher's Note

All claims expressed in this article are solely those of the authors and do not necessarily represent those of their affiliated organizations, or those of the publisher, the editors and the reviewers. Any product that may be evaluated in this article, or claim that may be made by its manufacturer, is not guaranteed or endorsed by the publisher.
