# Supplementary material for: Improvements in High-Density Lipoprotein Quantity and Quality Contribute to the Cardiovascular Benefits by Anti-tumor Necrosis Factor Therapies in Rheumatoid Arthritis: A Systemic Review and Meta-Analysis
Source: Front Cardiovasc Med. 2021 Oct 29;8:765749. doi: 10.3389/fcvm.2021.765749 (PMC8585789; doi:10.3389/fcvm.2021.765749)
Supplement: Supplementary file 1 [file Data_Sheet_1.docx]

Supplementary Material

# Supplementary materials 1

## Search strategy

We searched for English-language literature published on MEDLINE, Embase, and Cochrane to Cochrane Central Register of Controlled Trials (CENTRAL) updated to January 2021 using a combination of synonyms for the following keywords: 1) rheumatoid arthritis; 2) lipids 3) anti-TNF therapies. Search strategy in detail is as follows:

***1) MEDLINE***

(((((((((((cholesterol) OR LDL) OR HDL) OR triglyceride*) OR atherogenic index) OR apolipoprotein*) OR lipid*) OR dyslipidemia) OR hyperlipidemia)) AND (((((((((((((((((((((((infliximab) OR etanercept) OR adalimumab) OR certolizumab) OR golimumab) OR anti-TNF) OR anti-TNF alpha) OR tumor necrosis factor block*) OR tumor necrosis factor alpha block*) OR TNF block*) OR TNF alpha block*) OR tumor necrosis factor inhibit*) OR tumor necrosis factor alpha inhibit*) OR TNF inhibit*) OR TNF alpha inhibit*) OR tumor necrosis factor antibod*) OR tumor necrosis factor alpha antibod*) OR TNF antibod*) OR TNF alpha antibod*) OR tumor necrosis factor antagonist*) OR tumor necrosis factor alpha antagonist*) OR TNF antagonist*) OR TNF alpha antagonist*)) AND ((("arthritis rheumatoid"[Title/Abstract]) OR "rheumatoid arthritis"[Title/Abstract]) OR "rheumatoid, arthritis"[MeSH Terms])

***2)*** ***CENTRAL***

(((tumor necrosis factor alpha block*) OR (tumor necrosis factor alpha inhibit*) OR (tumor necrosis factor alpha antibod*) OR (tumor necrosis factor alpha antagonist*) OR (infliximab) OR (etanercept) OR (adalimumab) OR (certolizumab) OR (golimumab) OR (anti-TNF) OR (tumor necrosis factor block*) OR (tumor necrosis factor inhibit*) OR (tumor necrosis factor antibod*) OR (tumor necrosis factor antagonist*) OR (TNF block*) OR (TNF inhibit*) OR (TNF antibod*) OR (TNF antagonist*) OR (TNF alpha block*) OR (TNF alpha inhibit*) OR (TNF alpha antibod*) OR (TNF alpha antagonist*))) AND (((cholesterol) OR (LDL) OR (HDL) OR (triglyceride) OR (atherogenic index) OR (apolipoprotein) OR (lipid) OR (dyslipidemia) OR (hyperlipidemia))) AND (("rheumatoid arthritis")) in All Text - (Word variations have been searched)

***3) Embase.com***

('tumor necrosis factor alpha block*' OR 'tumor necrosis factor alpha inhibit*' OR 'tumor necrosis factor alpha antibod*' OR 'tumor necrosis factor alpha antagonist*' OR infliximab OR etanercept OR adalimumab OR certolizumab OR golimumab OR 'anti tnf' OR 'tumor necrosis factor block*' OR 'tumor necrosis factor inhibit*' OR 'tumor necrosis factor antibod*' OR 'tumor necrosis factor antagonist*' OR 'tnf block*' OR 'tnf inhibit*' OR 'tnf antibod*' OR 'tnf antagonist*' OR 'tnf alpha block*' OR 'tnf alpha inhibit*' OR 'tnf alpha antibod*' OR 'tnf alpha antagonist*') AND (cholesterol OR ldl OR hdl OR triglyceride OR 'atherogenic index' OR apolipoprotein OR lipid OR dyslipidemia OR hyperlipidemia) AND 'rheumatoid arthritis'

# Supplementary Figures and Tables

## Supplementary Figures


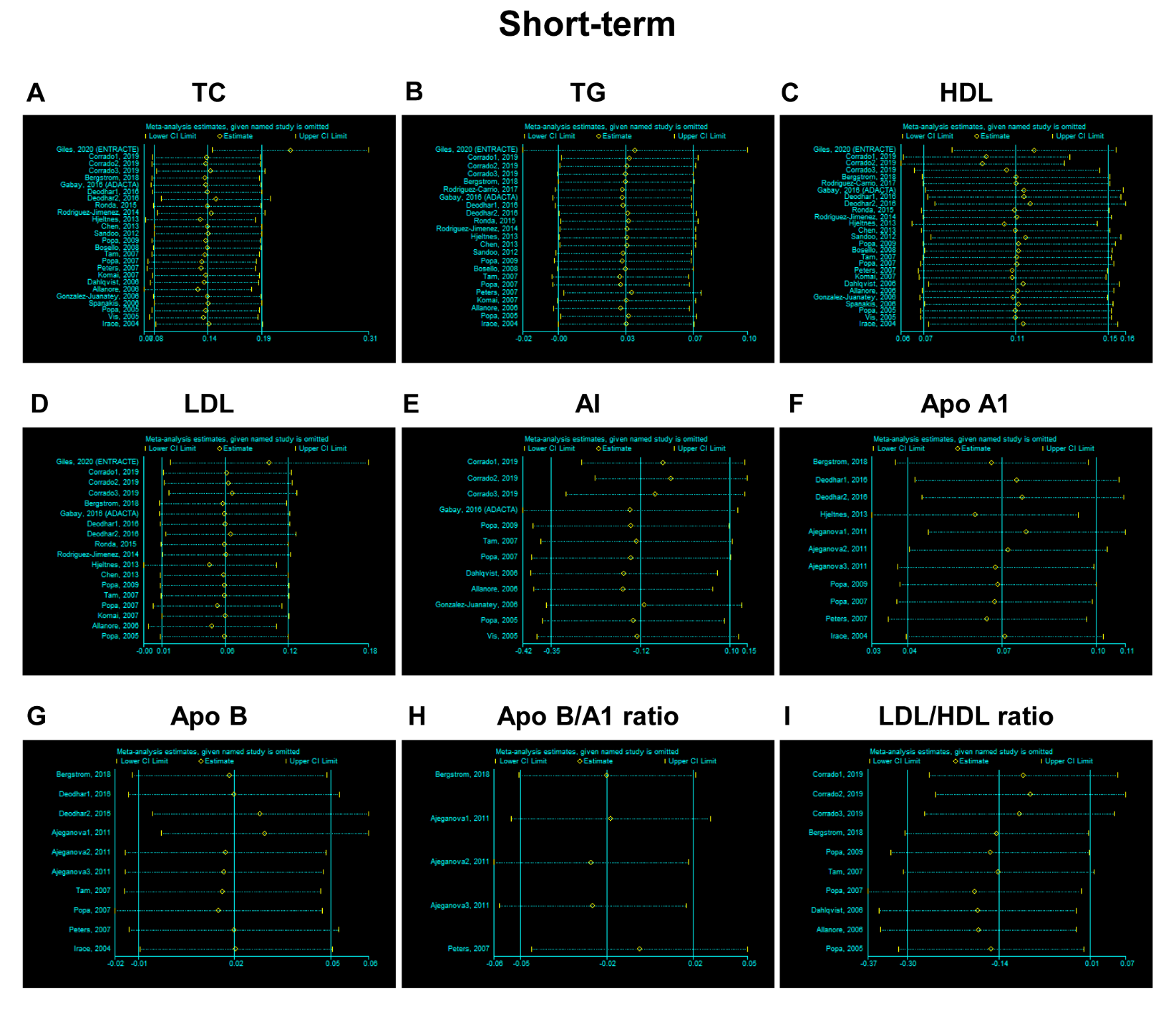


**Supplementary Figure 1.** Sensitivity analysis of meta-analyses of short-term changes of (**A**) TC, (**B**) TG, (**C**) HDL, (**D**) LDL, (**E**) AI, (**F**) Apo A1, (**G**) Apo B, (**H**) Apo B/Apo A1 ratio, and (**I**) LDL/HDL ratio. Abbreviations: TC, total cholesterol; TG, triglyceride; HDL, high-density lipoprotein; LDL, low-density lipoprotein; AI, atherogenic index; Apo A1, apolipoprotein A1; Apo B, apolipoprotein B.


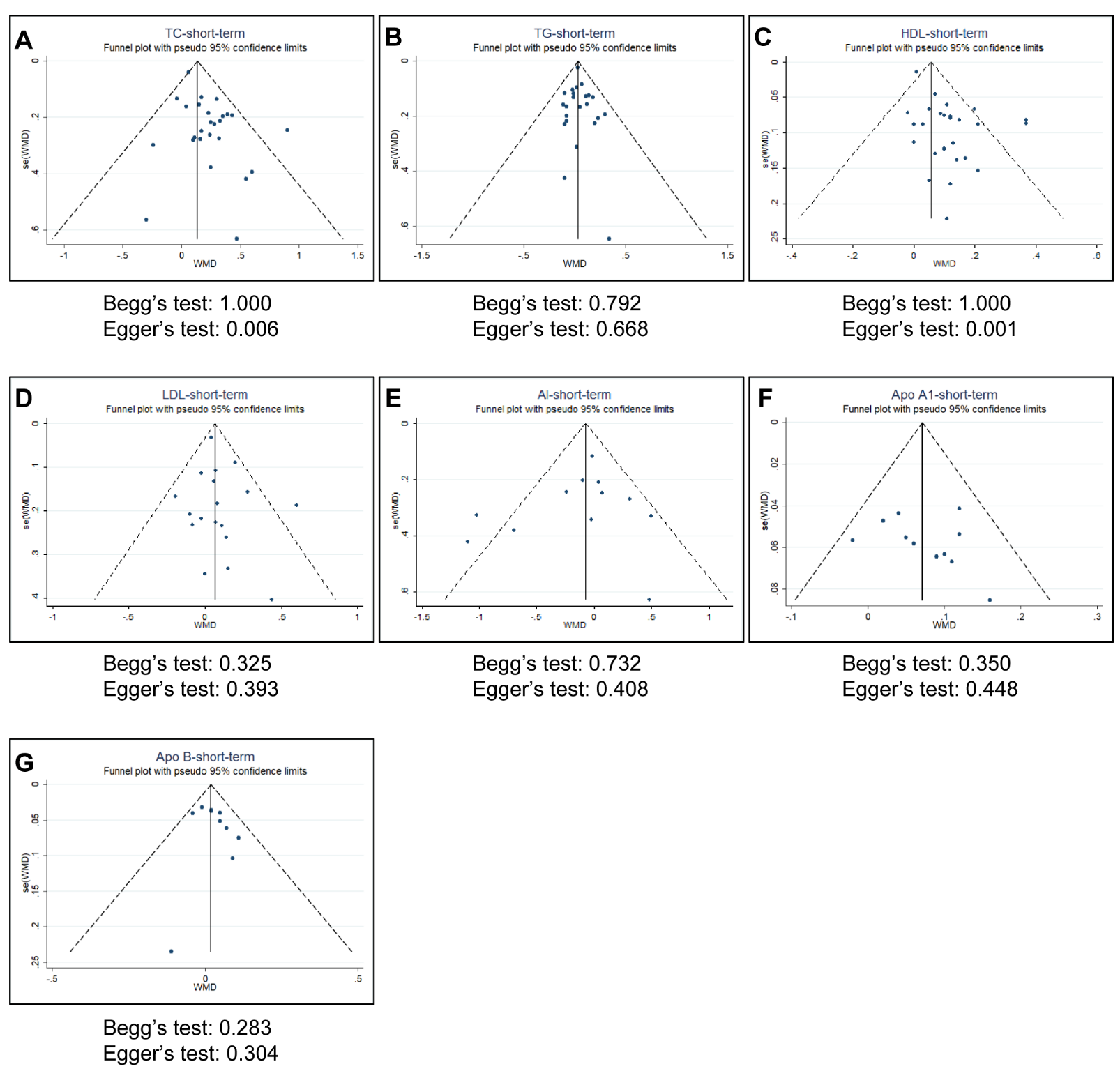


**Supplementary Figure 2.** Funnel plots of meta-analyses of short-term changes of (**A**) TC, (**B**) TG, (**C**) HDL, (**D**) LDL, (**E**) AI, (**F**) Apo A1, and (**G**) Apo B. Abbreviations: TC, total cholesterol; TG, triglyceride; HDL, high-density lipoprotein; LDL, low-density lipoprotein; AI, atherogenic index; Apo A1, apolipoprotein A1; Apo B, apolipoprotein B.


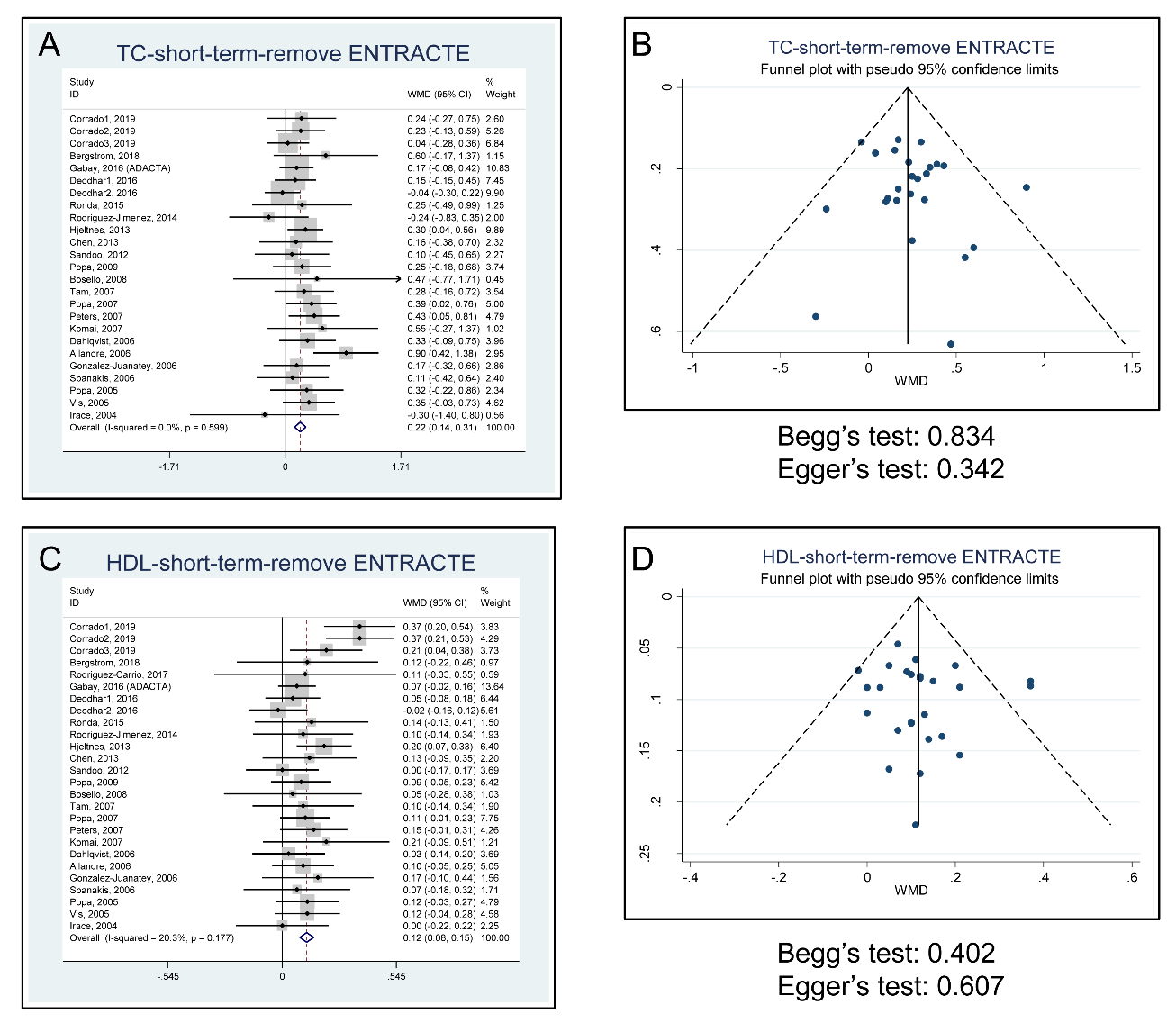


**Supplementary Figure 3.** Analysis of the asymmetries of the funnel plots for TC and HDL changes in short-term. (**A**) Forest plot and (**B**) funnel plot of meta-analysis of TC changes in short-term after removing the ENTRACTE trial. (**C**) Forest plot and (**D**) funnel plot of meta-analysis of TC changes in short-term after removing the ENTRACTE trial. Abbreviations: TC, total cholesterol; HDL, high-density lipoprotein.


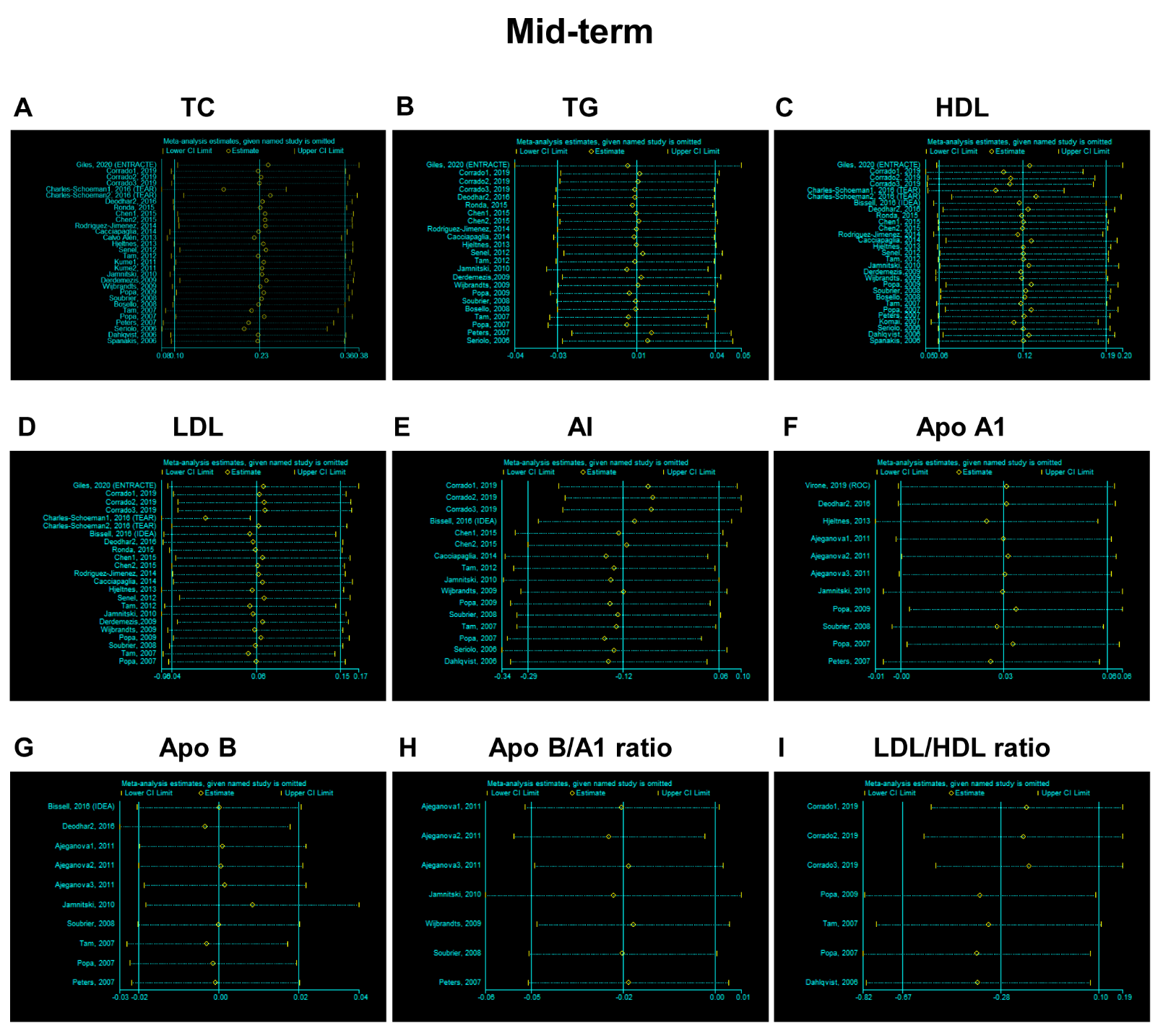


**Supplementary Figure 4.** Sensitivity analysis of meta-analyses of mid-term changes of (**A**) TC, (**B**) TG, (**C**) HDL, (**D**) LDL, (**E**) AI, (**F**) Apo A1, (**G**) Apo B, (**H**) Apo B/Apo A1 ratio, and (**I**) LDL/HDL ratio. Abbreviations: TC, total cholesterol; TG, triglyceride; HDL, high-density lipoprotein; LDL, low-density lipoprotein; AI, atherogenic index; Apo A1, apolipoprotein A1; Apo B, apolipoprotein B.


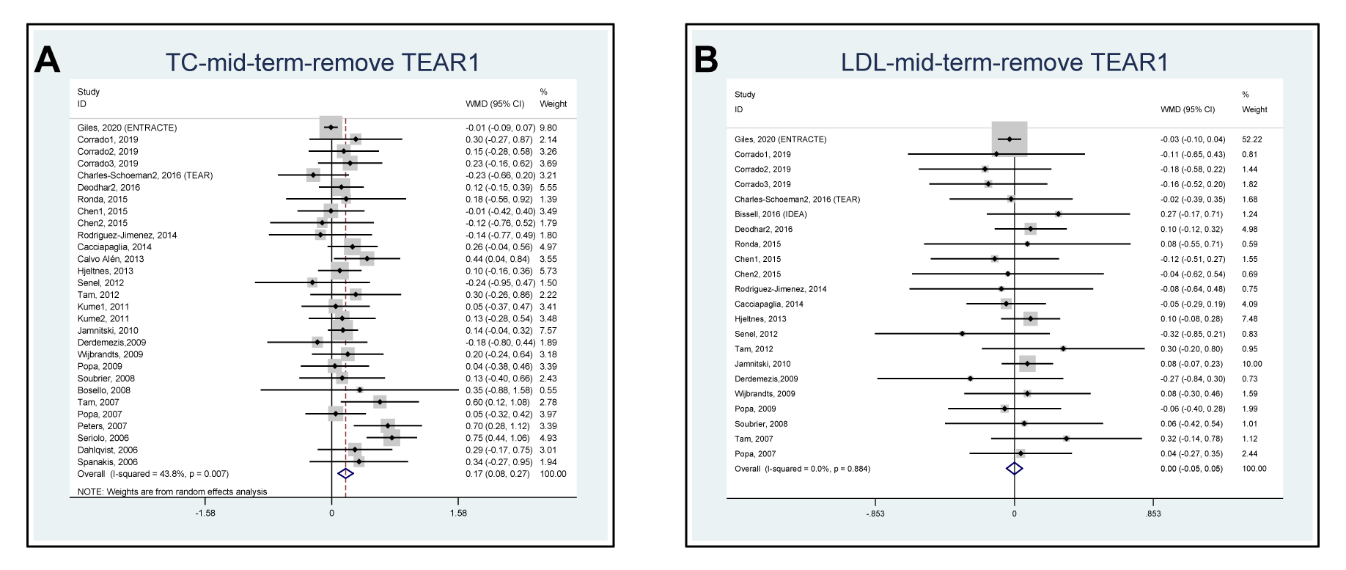


**Supplementary Figure 5.** Analysis of the effects of the TEAR1 trial on meta-analyses of changes in mid-term TC and LDL. Forest plots of meta-analyses of (**A**) TC and (**B**) LDL changes in mid-term after removing the TEAR1 trial. Abbreviations: TC, total cholesterol; LDL, low-density lipoprotein.


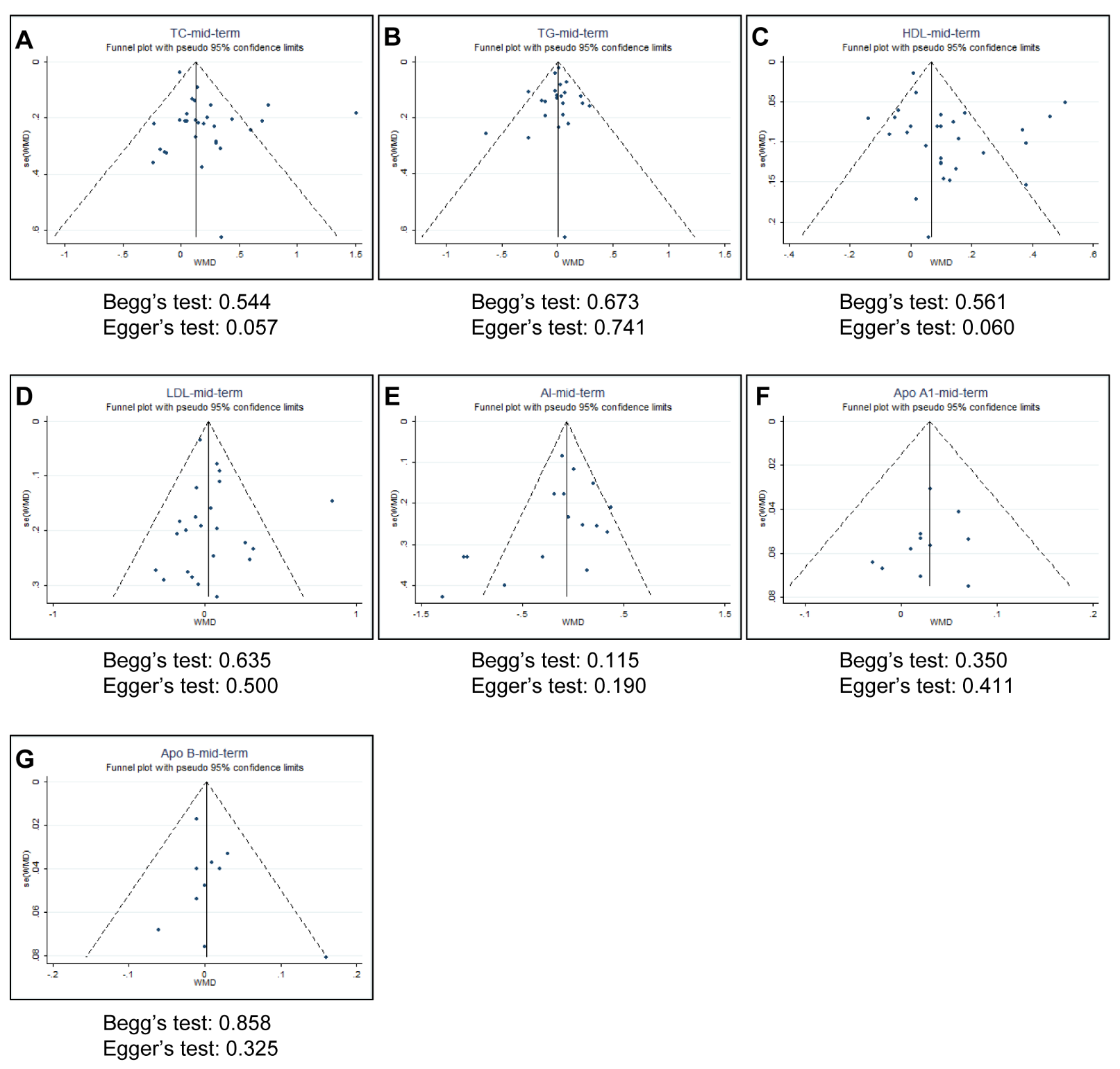


**Supplementary Figure 6.** Funnel plots of meta-analyses of mid-term changes of (**A**) TC, (**B**) TG, (**C**) HDL, (**D**) LDL, (**E**) AI, (**F**) Apo A1, and (**G**) Apo B. Abbreviations: TC, total cholesterol; TG, triglyceride; HDL, high-density lipoprotein; LDL, low-density lipoprotein; AI, atherogenic index; Apo A1, apolipoprotein A1; Apo B, apolipoprotein B.


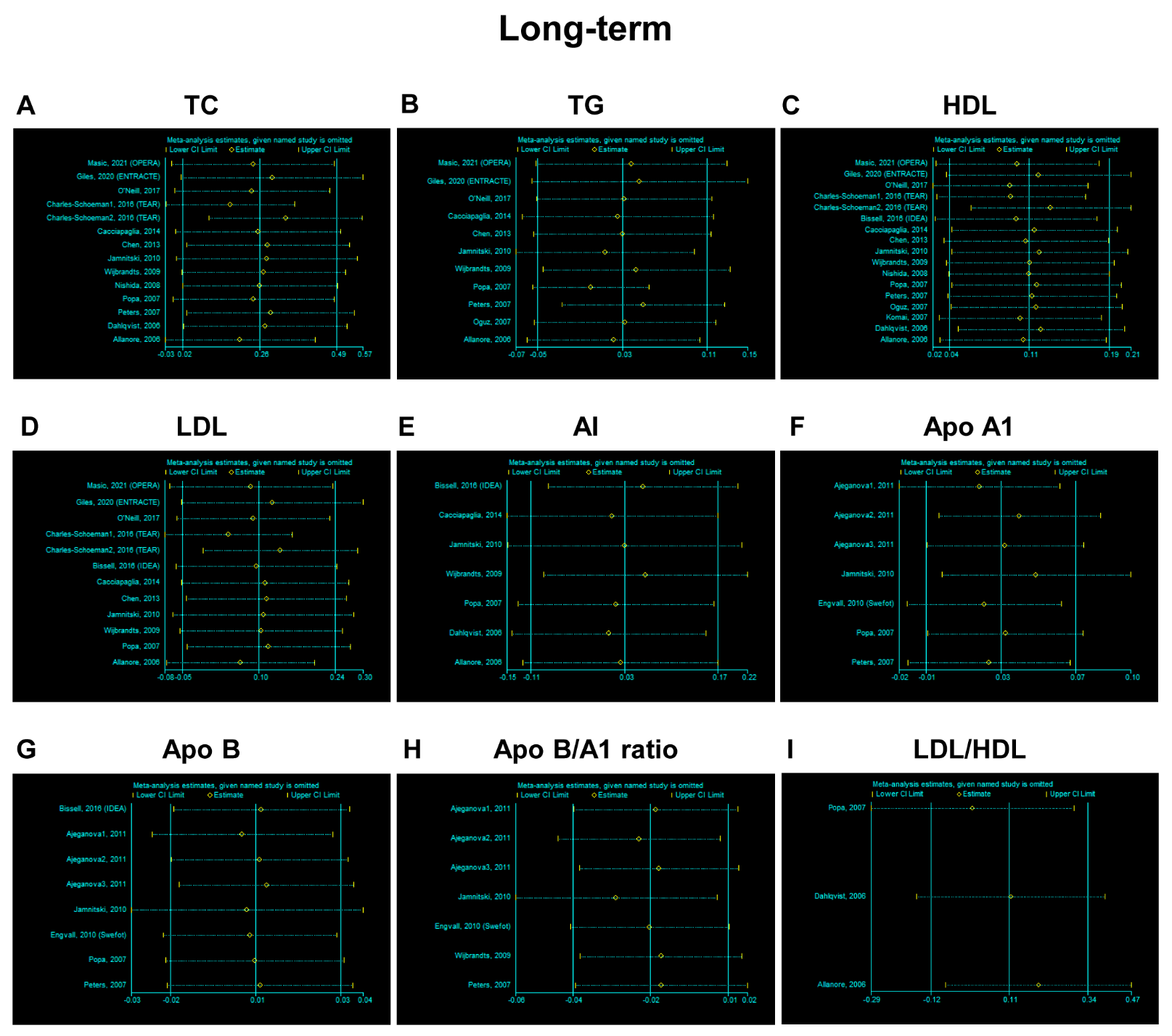


**Supplementary Figure 7.** Sensitivity analysis of meta-analyses of long-term changes of (**A**) TC, (**B**) TG, (**C**) HDL, (**D**) LDL, (**E**) AI, (**F**) Apo A1, (**G**) Apo B, (**H**) Apo B/Apo A1 ratio, and (**I**) LDL/HDL ratio. Abbreviations: TC, total cholesterol; TG, triglyceride; HDL, high-density lipoprotein; LDL, low-density lipoprotein; AI, atherogenic index; Apo A1, apolipoprotein A1; Apo B, apolipoprotein B.


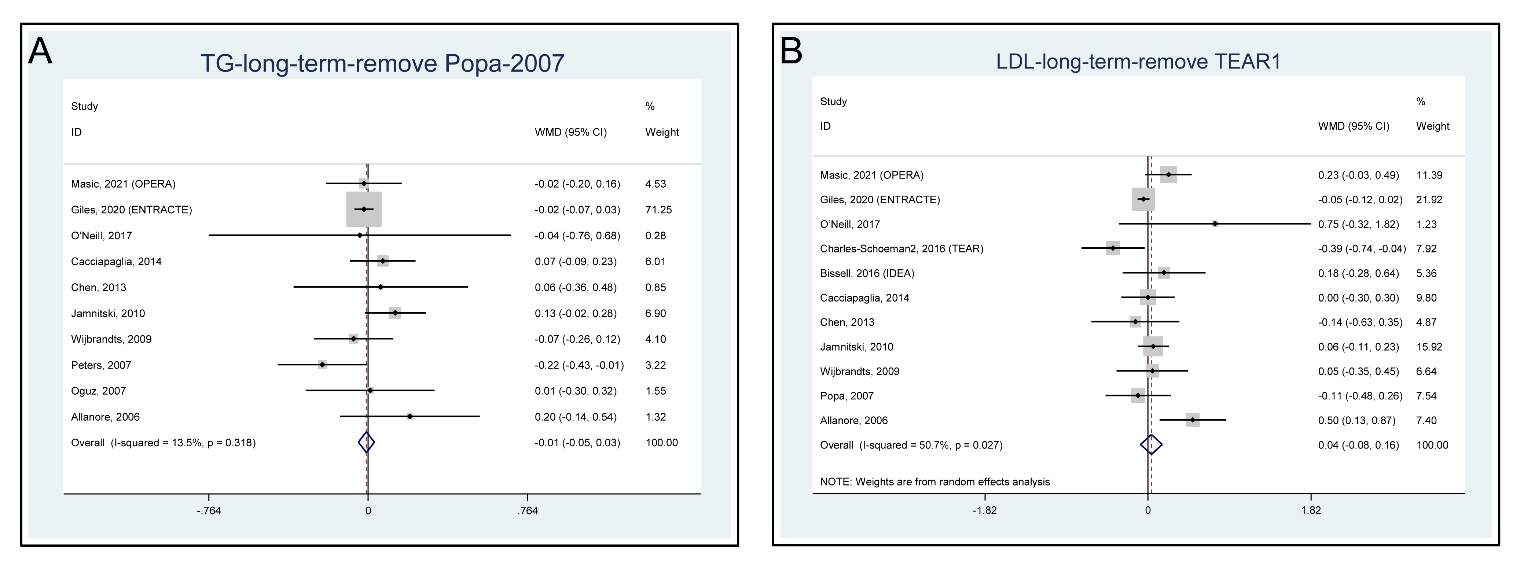


**Supplementary Figure 8.** (**A**) Forest plot of meta-analysis of TG changes in the long-term after removing the Popa-2007 study. (**B**) Forest plot of meta-analysis of LDL changes in the long-term after removing the TEAR1 study. Abbreviations: TG, triglyceride; LDL, low-density lipoprotein.


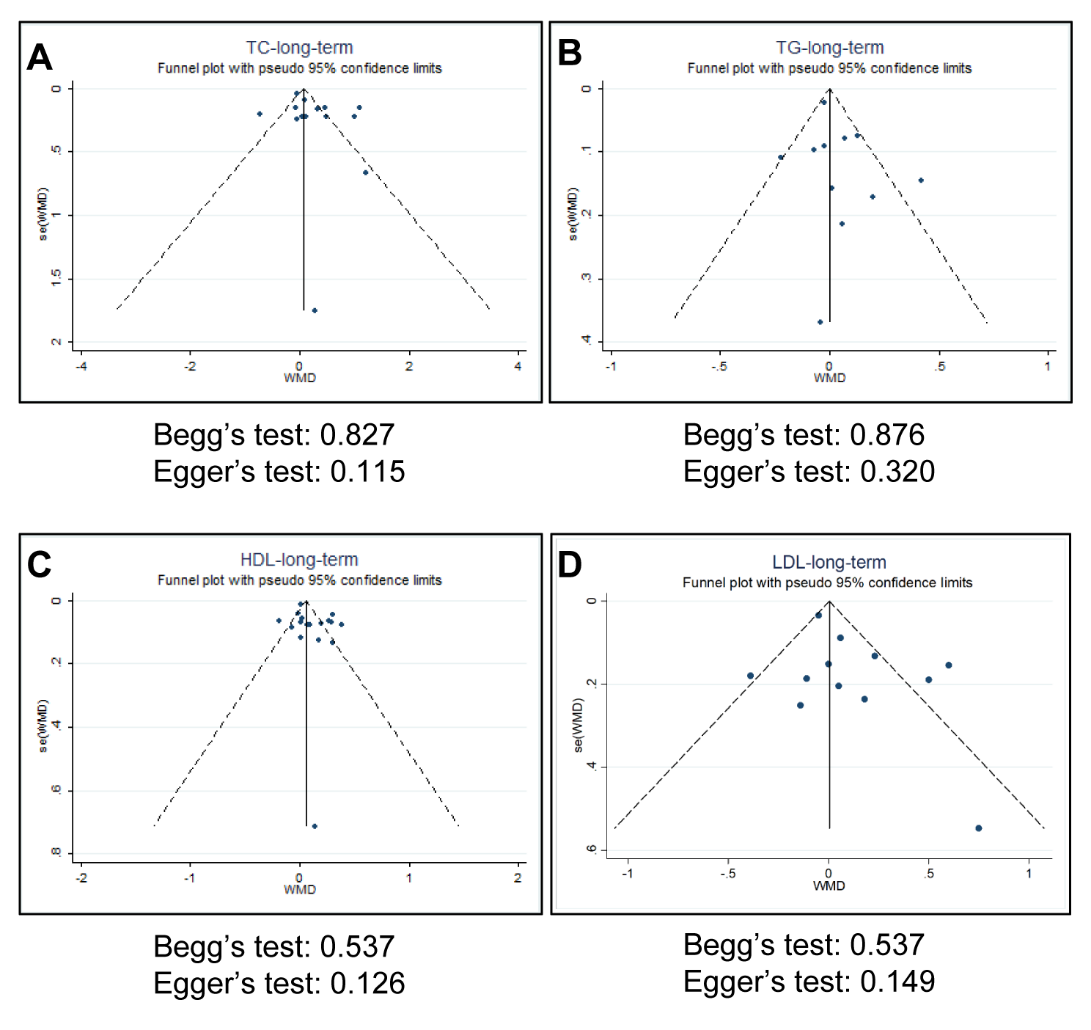


**Supplementary Figure 9.** Funnel plots of meta-analyses of long-term changes of (**A**) TC, (**B**) TG, (**C**) HDL, and (**D**) LDL. Abbreviations: TC, total cholesterol; TG, triglyceride; HDL, high-density lipoprotein; LDL, low-density lipoprotein.

## Supplementary Tables

**Supplementary Table 1.** Concomitant drug use at baseline.

| **Study** | **Corticosteroids use at baseline** | **MTX or cDMARDs use at baseline** | **Statins or other lipid-lowering drugs use at baseline** |
| --- | --- | --- | --- |
| Masic, 2021 (OPERA) | none | 100% | 10.50% |
| Giles, 2020 (ENTRACTE) | 26% | 71% | - |
| Corrado1, 2019 | prednisone <10 mg/day | 100% | none or <6m |
| Corrado2, 2019 | prednisone <10 mg/day | 100% | none or <6m |
| Corrado3, 2019 | prednisone <10 mg/day | 100% | none or <6m |
| Virone, 2019 (ROC) | 47.9% (oral equivalent of prednisone ≤15 mg/day) | 100% | - |
| Bergstrom, 2018 | 64% | 57% | none |
| Rodriguez-Carrio, 2017 | - | previous cDMARDs failure | - |
| O’Neill, 2017 | none or stable prednisolone 10 mg/day for 4 weeks | 100% (MTX 12.5-17.5 mg/week) | - |
| Gabay, 2016 (ADACTA) | 57% | None (wash out) | - |
| Charles-Schoeman1, 2016 (TEAR) | 42.86% (prednisone＜10 mg/day) | minimal or no prior use | 12.65% |
| Charles-Schoeman2, 2016 (TEAR) |  | minimal or no prior use |  |
| Bissell, 2016 (IDEA) | none | none | none |
| Deodhar1, 2016 | 65.40% | 100% | 20.20% |
| Deodhar2, 2016 | 68.90% | 100% | 24.50% |
| Ronda, 2015 | 27.30% | 100% (MTX 18 ± 31.9 mg/week) | 18.20% |
| Chen1, 2015 | 100% (6.3 ± 2.5 mg/day) | 100% | - |
| Chen2, 2015 | 100% (6.9 ± 1.9 mg/day) | 100% | - |
| Rodriguez-Jimenez, 2014 | none or prednisone equivalent <10mg/day | none | none |
| Cacciapaglia, 2014 | 56.30% | 61.30% | - |
| Calvo Alén, 2013 | - | - | - |
| Hjeltnes, 2013 | 30% | 100% | 20% |
| Chen, 2013 | - | 100% | - |
| Sandoo, 2012 | 26% | 70% | 17% |
| Senel, 2012 | <10 mg/day | 100% | - |
| Tam, 2012 | 30% (prednisolone <10 mg/day) | 100% | none |
| Ajeganova1, 2011 | 33.3% [median/IQR: 5 (5-7.5) mg/day] | 77.80% | 3.70% |
| Ajeganova2, 2011 |  |  |  |
| Ajeganova3, 2011 |  |  |  |
| Kume1, 2011 | none | none | - |
| Kume2, 2011 | none | none | - |
| Jamnitski, 2010 | 29% | 76% | 13% |
| Engvall, 2010 (Swefot) | 11.10% | 100% | 11.10% |
| Derdemezis, 2009 | 100% (prednisone ≤7.5 g/day) | 100% (MTX 0.2 mg/kg/week) | none |
| Wijbrandts, 2009 | 32% (prednisone 7.8 ± 2.4 mg/day) | 100% | 12% |
| Popa, 2009 | none | - | none |
| Soubrier, 2008 | 69.0% (8 ± 3 mg/day) | 69.00% | - |
| Nishida, 2008 | 3.3 ± 0.6 mg/day | 100% | - |
| Bosello, 2008 | 100% (prednisone 5 mg/day) | 100% (MTX 15–20 mg/week) | - |
| Tam, 2007 | 5.3% (prednisolone 7.5 mg/day) | 100% | none |
| Popa, 2007 | 16.40% | 61.80% | none |
| Peters, 2007 | 44% (prednisone 8.3 mg/day) | 96% (MTX 15 mg/week) | 5% |
| Oguz, 2007 | 100% (prednisolone 8.2 mg/day) | 85.7% (MTX 17.5 mg/day) | - |
| Komai, 2007 | 100% (prednisolone ≤10 mg/day) | 100% (MTX ≤10 mg/week) | - |
| eriolo, 2006 | 100% (prednisone <10 mg/day) | 100% (MTX 10 mg/week) | - |
| Dahlqvist, 2006 | 53.80% | 78.80% | none |
| Allanore, 2006 | 100% [prednisone mean (range): 7.4 (2.5–10) mg/day] | 95% (MTX 13 mg/week) | none |
| Gonzalez-Juanatey, 2006 | 100% [prednisone median (range): 7.5 (5-10) mg/day] | 100% (MTX 15 mg/week) | none |
| Spanakis, 2006 | 25.0% (prednisone mean 7 mg/day) | 87.5% (MTX 13 ± 3.5 mg/week) | none |
| Popa, 2005 | - | wash out for at least 3 weeks | - |
| Vis, 2005 | 46% [prednisone mean (range): 10.6 (2.5–30) mg/day] | 90% (MTX 15.8 mg/week) | - |
| Irace, 2004 | 100% (methylprednisolone <10 mg/day) | 100% (MTX <25 mg/week) | none |

Data are mean (± SD) unless otherwise specified. Abbreviations: cDMARDs, conventional disease-modifying antirheumatic drugs; MTX, methotrexate.

**Supplementary Table 2.** Clinical trial registration numbers for RCTs.

| **RCT** | **Clinical trial registration numbers** |
| --- | --- |
| Masic, 2021 (OPERA) (9) | NCT00660647 |
| Giles, 2020 (ENTRACTE) (10) | NCT01331837 |
| Virone, 2019 (ROC) (11) | NCT01000441 |
| O’Neill, 2017 (12) | Unavailable |
| Gabay, 2016 (ADACTA) (13) | NCT01119859 |
| Charles-Schoeman, 2016 (TEAR) (14) | NCT00259610 |
| Bissell, 2016 (IDEA) (15) | Eudract-2005-005013-37; ISRCTN48638981 |
| Deodhar, 2016 (16) | NCT01313208 |
| Tam, 2012 (17) | Unavailable |
| Kume, 2011 (18) | Unavailable |
| Engvall, 2010 (Swefot) (19) | ISRCTN39045408 |

Abbreviations: RCT, randomized controlled trial.

**Supplementary Table 3.** Quality assessment of the included trials.

| **Study** | **Study design** | | | **Represen-tativeness** | **Detection bias** | **Confounding factors** | | | | | | | **Adequacy of follow up** | | **Quality score** |
| --- | --- | --- | --- | --- | --- | --- | --- | --- | --- | --- | --- | --- | --- | --- | --- |
|  |  |  |  |  |  | **Listed at baseline** | | | **Previous Anti-TNFα therapy** | **Controlled during treatment** | | |  |  |  |
|  | **Study designed to evaluate lipid levels** | **Selection criteria clearly described** | **Consecutive patients selection** | **Representativeness**  **of study population** | **Blindness of**  **outcome assessment** | **MTX or other cDMARDs use** | **Steroids use** | **Statins or other lipid-lowering use** |  | **MTX or other cDMARDs use** | **Steroids use** | **Statins or other lipid-lowering use** | **Dropout rate^5^** | **Withdraws explained** |  |
| Masic, 2021 (OPERA) | * | * | * | no^1^ | * | * | * | * | 0 | * | * | NA | 9.0% | * | 12 |
| Giles, 2020 (ENTRACTE) | no | * | NA | no^2^ | * | * | * | NA | NA | NA | NA | NA | 4.3% | * | 6 |
| Corrado1, 2019 | * | * | * | * | * | * | * | * | 0 | * | * | NA | 0 | / | 14 |
| Corrado2, 2019 | * | * | * | * | * | * | * | * | 0 | * | * | NA | 0 | / | 14 |
| Corrado3, 2019 | * | * | * | * | * | * | * | * | 0 | * | * | NA | 0 | / | 14 |
| Virone, 2019 (ROC) | no | * | * | no^3^ | * | * | * | NA | 100% | * | * | NA | 10.0% | * | 9 |
| Bergstrom，2018 | * | * | * | * | * | * | * | * | 0 | * | * | * | 0 | / | 14 |
| Rodriguez-Carrio, 2017 | * | * | NA | * | * | * | NA | NA | 0 | NA | NA | NA | 0 | / | 8 |
| O’Neill, 2017 | no | * | NA | * | * | * | * | NA | 0 | * | * | NA | 0 | / | 10 |
| Gabay, 2016 (ADACTA) | * | * | NA | * | * | * | * | NA | 0 | * | * | NA | 11.6% | * | 10 |
| Charles-Schoeman1, 2016 (TEAR) | no | * | NA | no^1^ | * | * | * | * | 0 | * | * | NA | 31.1% | * | 9 |
| Charles-Schoeman2, 2016 (TEAR) | no | * | NA | no^1^ | * | * | * | * | 0 | * | * | NA | NA | no | 8 |
| Bissell, 2016 (IDEA) | no | * | * | no^1^ | * | * | * | * | 0 | * | * | no | 20.0% | * | 10 |
| Deodhar1, 2016 | no | * | NA | * | * | * | * | * | 0 | * | NA | NA | 6.1% | * | 10 |
| Deodhar2, 2016 | no | * | NA | * | * | * | * | * | 0 | * | NA | NA | 7.5% | * | 10 |
| Ronda, 2015 | no | * | NA | * | * | * | * | * | 0 | * | NA | NA | 0 | / | 10 |
| Chen1, 2015 | * | * | * | * | * | * | * | NA | 0 | * | * | NA | 0 | / | 12 |
| Chen2, 2015 | * | * | * | * | * | * | * | NA | 0 | * | * | NA | 0 | / | 12 |
| Rodriguez-Jimenez，2014 | * | * | * | * | * | * | * | * | 0 | * | * | * | 0 | / | 14 |
| Cacciapaglia，2014 | * | * | NA | * | * | * | * | NA | NA | NA | NA | NA | NA | no | 6 |
| Calvo Alén, 2013 | * | * | NA | * | * | NA | NA | NA | 0 | NA | NA | NA | 0 | / | 7 |
| Hjeltnes, 2013 | * | * | * | * | * | * | * | * | 0 | * | NA | NA | 13.5% | * | 11 |
| Chen, 2013 | no | * | NA | * | * | * | NA | NA | NA | * | * | NA | 0 | / | 8 |
| Sandoo, 2012 | * | * | * | * | * | * | * | * | 0 | * | * | * | 0 | / | 14 |
| Senel, 2012 | * | * | * | * | * | * | * | NA | NA | * | * | NA | 15.8% | * | 10 |
| Tam, 2012 | no | * | NA | no^1^ | * | * | * | * | 0 | * | * | * | 0 | / | 11 |
| Ajeganova1, 2011 | * | * | * | * | * | * | * | * | 0 | * | * | * | 0 | / | 14 |
| Ajeganova2, 2011 | * | * | * | * | * | * | * | * | 0 | * | * | * | 20.0% | no | 12 |
| Ajeganova3, 2011 | * | * | * | * | * | * | * | * | 0 | * | * | * | 33.3% | no | 12 |
| Kume1, 2011 | * | * | NA | * | * | * | * | NA | 0 | * | * | NA | 4.8% | no | 10 |
| Kume2, 2011 | * | * | NA | * | * | * | * | NA | 0 | * | * | NA | 9.5% | no | 10 |
| Jamnitski, 2010 | * | * | * | * | * | * | * | * | 31% | * | * | * | 34.5% | * | 12 |
| Engvall, 2010 (Swefot) | no | * | * | no^1^ | * | * | * | * | 0 | * | * | NA | 38.9% | * | 10 |
| Derdemezis, 2009 | no | * | * | no^4^ | * | * | * | * | 0 | * | * | * | no | / | 12 |
| Wijbrandts, 2009 | * | * | NA | no^1^ | * | * | * | * | NA | * | * | NA | 12.0% | * | 9 |
| Popa, 2009 | * | * | * | * | * | no | * | * | 0 | * | * | * | 0 | / | 13 |
| Soubrier, 2008 | * | * | NA | * | * | * | * | NA | NA | NA | * | NA | 0 | / | 9 |
| Nishida, 2008 | no | NA | NA | * | * | * | * | NA | NA | NA | * | NA | NA | no | 5 |
| Bosello, 2008 | no | * | * | * | * | * | * | NA | NA | * | * | NA | 0 | / | 10 |
| Tam, 2007 | * | * | * | no^4^ | * | * | * | * | NA | * | * | * | 0 | / | 12 |
| Popa, 2007 | * | * | * | * | * | * | * | * | 0 | * | * | * | 43.6% | no | 12 |
| Peters, 2007 | * | * | * | * | * | * | * | * | NA | * | no | * | 25.9% | * | 11 |
| Oguz, 2007 | no | * | NA | * | * | * | * | NA | NA | NA | NA | NA | 0 | / | 7 |
| Komai, 2007 | no | * | NA | * | * | * | * | NA | NA | NA | NA | NA | 0 | / | 7 |
| Seriolo, 2006 | * | * | * | no^4^ | * | * | * | NA | NA | * | * | NA | 0 | / | 10 |
| Dahlqvist, 2006 | * | * | * | * | * | * | * | * | NA | NA | * | * | 21.2% | * | 11 |
| Allanore, 2006 | * | * | * | * | * | * | * | * | NA | NA | NA | * | 5.1% | * | 11 |
| Gonzalez-Juanatey, 2006 | no | * | * | * | * | * | * | * | 100% | * | * | * | 0 | / | 12 |
| Spanakis, 2006 | * | * | * | * | * | * | * | * | NA | * | * | * | 0 | / | 13 |
| Popa, 2005 | * | * | NA | * | * | * | * | NA | NA | * | * | NA | 0 | / | 10 |
| Vis, 2005 | * | * | * | * | * | * | * | NA | NA | NA | * | NA | 0 | / | 10 |
| Irace, 2004 | * | * | NA | * | * | * | * | * | NA | * | * | * | 0 | / | 12 |

* indicates low risk.

1. Patients with disease duration (mean or median) <6 months, which are considered as early RA;

2. Patients with ≥1 traditional CVD risk factor or history of a CVD event;

3. Patients with insufficient response to anti-TNF therapies;

4. All female patients;

5. If the study is a secondary analysis of a trial, dropout rates were obtained from the original trial; a dropout rate <10% is considered as no biases.

Abbreviations: cDMARDs, conventional disease-modifying antirheumatic drugs; MTX, methotrexate; NA, not available.

**Supplementary Table 4.** Subgroup analysis of changes in TC after anti-TNF therapies in short-, mid-, and long-term.

|  | **TC-short-term** | | | | |  | **TC-mid-term** | | | | |  | **TC-long-term** | | | | |
| --- | --- | --- | --- | --- | --- | --- | --- | --- | --- | --- | --- | --- | --- | --- | --- | --- | --- |
|  | **Studies (n)** | **Patients (n)** | **WMD (95% CI)** | **Heterogeneity *I*^2^, *p*-value** | ***p*-value*** |  | **Studies (n)** | **Patients (n)** | **WMD (95% CI)** | **Heterogeneity *I*^2^, *p*-value** | ***p*-value*** |  | **Studies (n)** | **Patients (n)** | **WMD (95% CI)** | **Heterogeneity *I*^2^, *p*-value** | ***p*-value*** |
| **In total** | 26 | 2530 | 0.14 (0.08, 0.19) | 15.8%, 0.236 |  |  | 30 | 2918 | 0.23 (0.10, 0.38) | 73.1%, 0.000 |  |  | 14 | 2639 | 0.26 (0.02, 0.29) | 86.9%, 0.000 |  |
| **1. quality score** |  |  |  |  | 0.029 |  |  |  |  |  | 0.460 |  |  |  |  |  | 0.063 |
| <9 | 3 | 1576 | 0.07 (-0.01, 0.14) | 0.0%, 0.478 |  |  | 4 | 1744 | 0.10 (-0.13, 0.34) | 64.2%, 0.039 |  |  | 5 | 1842 | -0.10 (-0.42, 0.23) | 73.8%, 0.004 |  |
| >=9 | 23 | 954 | 0.22 (0.14, 0.31) | 0.0%, 0.520 |  |  | 26 | 1174 | 0.25 (0.10, 0.40) | 68.5%, 0.000 |  |  | 9 | 797 | 0.44 (0.13, 0.74) | 83.9%, 0.000 |  |
| **2. study design** |  |  |  |  | 0.002 |  |  |  |  |  | 0.767 |  |  |  |  |  | 0.862 |
| RCT | 4 | 1890 | 0.07 (-0.00, 0.14) | 0.0%, 0.660 |  |  | 7 | 1954 | 0.27 (-0.11, 0.64) | 91.2%, 0.000 |  |  | 5 | 1883 | 0.32 (-0.26, 0.89) | 94.6%, 0.000 |  |
| Cohort | 22 | 640 | 0.28 (0.18, 0.37) | 0.0%, 0.752 |  |  | 23 | 964 | 0.23 (0.12, 0.33) | 28.1%, 0.104 |  |  | 9 | 756 | 0.23 (0.02, 0.44) | 60.6%, 0.009 |  |
| **3. RA duration** |  |  |  |  | 0.798 |  |  |  |  |  | 0.322 |  |  |  |  |  | 0.994 |
| <6 months | 0 | 0 | - | - |  |  | 4 | 315 | 0.45 (-0.38, 1.28) | 93.0%, 0.000 |  |  | 4 | 381 | 0.25 (-0.47, 0.98) | 94.0%, 0.000 |  |
| >=6 months | 22 | 2464 | 0.13 (0.07, 0.19) | 26.2%, 0.127 |  |  | 22 | 2541 | 0.18 (0.07, 0.30) | 54.2%, 0.001 |  |  | 10 | 2258 | 0.21 (0.01, 0.42) | 73.5%, 0.000 |  |
| unclear | 4 | 66 | 0.17 (-0.03, 0.37) | 0.0%, 0.772 |  |  | 4 | 62 | 0.20 (-0.03, 0.43) | 0.0%, 0.967 |  |  | 0 | 0 | - | - |  |
| **4. DAS28** |  |  |  |  | 0.382 |  |  |  |  |  | 0.539 |  |  |  |  |  | 0.632 |
| <=5.1 | 8 | 284 | 0.10 (-0.04, 0.24) | 0.0%, 0.787 |  |  | 7 | 271 | 0.17 (0.02, 0.32) | 0.0%, 0.919 |  |  | 2 | 91 | 0.54 (-0.20, 1.28) | 40.1%, 0.196 |  |
| >5.1 | 14 | 596 | 0.26 (0.14, 0.38) | 0.0%, 0.933 |  |  | 21 | 1076 | 0.27 (0.08, 0.46) | 74.9%, 0.000 |  |  | 10 | 951 | 0.18 (-0.13, 0.49) | 85.5%, 0.000 |  |
| unclear | 4 | 1650 | 0.10 (0.03, 0.17) | 78.3%, 0.003 |  |  | 2 | 1571 | -0.00 (-0.07, 0.07) | 0.0%, 0.431 |  |  | 2 | 1597 | 0.45 (-0.58, 1.48) | 95.2%, 0.000 |  |
| **5. age** |  |  |  |  | 0.860 |  |  |  |  |  | 0.512 |  |  |  |  |  | 0.892 |
| <55 years old | 11 | 413 | 0.21 (0.09, 0.33) | 4.9%, 0.398 |  |  | 18 | 925 | 0.26 (0.05, 0.47) | 76.7%, 0.000 |  |  | 10 | 952 | 0.28 (-0.05, 0.61) | 86.7%, 0.000 |  |
| >=55 years old | 14 | 2084 | 0.11 (0.05, 0.18) | 25.7%, 0.199 |  |  | 12 | 1993 | 0.14 (0.02, 0.26) | 34.8%, 0.112 |  |  | 4 | 1687 | 0.13 (-0.17, 0.43) | 68.4%, 0.023 |  |
| unclear | 1 | 33 | 0.32 (-0.22, 0.86) | - |  |  | 0 | 0 | - | - |  |  | 0 | 0 | - | - |  |
| **6. drug** |  |  |  |  | 0.004 |  |  |  |  |  | 0.794 |  |  |  |  |  | 0.793 |
| IFX | 12 | 446 | 0.36 (0.22, 0.49) | 0.0%, 0.683 |  |  | 10 | 346 | 0.26 (0.09, 0.43) | 16.9%, 0.288 |  |  | 6 | 351 | 0.43 (-0.02, 0.87) | 73.6%, 0.002 |  |
| ADA | 6 | 232 | 0.22 (0.04, 0.40) | 0.0%, 0.938 |  |  | 5 | 135 | 0.14 (-0.08, 0.35) | 0.0%, 0.924 |  |  | 2 | 136 | 0.34 (-0.00, 0.68) | 41.8%, 0.190 |  |
| ETN | 6 | 1799 | 0.05 (-0.02, 0.12) | 0.0%, 0.846 |  |  | 10 | 2245 | 0.16 (-0.10, 0.42) | 87.2%, 0.000 |  |  | 5 | 2072 | 0.09 (-0.32, 0.50) | 93.6%, 0.000 |  |
| mixed | 2 | 53 | 0.26 (0.03, 0.50) | 0.0%, 0.520 |  |  | 5 | 192 | 0.34 (0.09, 0.60) | 64.2%, 0.025 |  |  | 1 | 80 | 0.33 (0.00, 0.66) | - |  |
| **7. baseline TC** |  |  |  |  | 0.190 |  |  |  |  |  | 0.032 |  |  |  |  |  | 0.230 |
| <5.2 mmol/l | 18 | 2278 | 0.11 (0.05, 0.17) | 24.9%, 0.162 |  |  | 17 | 2437 | 0.35 (0.15, 0.55) | 84.2%, 0.000 |  |  | 11 | 2428 | 0.35 (0.08, 0.61) | 87.7%, 0.000 |  |
| >=5.2 mmol/l | 8 | 252 | 0.30 (0.14, 0.45) | 0.0%, 0.926 |  |  | 13 | 481 | 0.06 (-0.06, 0.18) | 0.0%, 0.948 |  |  | 3 | 211 | -0.05 (-0.76, 0.65) | 87.8%, 0.000 |  |

***** *p*-value for meta regression. Abbreviations: TC, total cholesterol; RCT, randomized controlled study; DAS28, 28 joint disease activity score; IFX, Infliximab; ADA, Adalimumab; ETN, Etanercept.

**Supplementary Table 5.** Subgroup analysis of changes in TG after anti-TNF therapies in short-, mid-, and long-term.

|  | **TG-short-term** | | | | |  | **TG-mid-term** | | | | |  | **TG-long-term** | | | | |
| --- | --- | --- | --- | --- | --- | --- | --- | --- | --- | --- | --- | --- | --- | --- | --- | --- | --- |
|  | **Studies (n)** | **Patients (n)** | **WMD (95% CI)** | **Heterogeneity *I*^2^, *p*-value** | ***p*-value*** |  | **Studies (n)** | **Patients (n)** | **WMD (95% CI)** | **Heterogeneity *I*^2^, *p*-value** | ***p*-value*** |  | **Studies (n)** | **Patients (n)** | **WMD (95% CI)** | **Heterogeneity *I*^2^, *p*-value** | ***p*-value*** |
| **In total** | 23 | 2390 | 0.03 (-0.00, 0.07) | 0.0%, 0.979 |  |  | 23 | 2536 | 0.01 (-0.03, 0.04) | 14.0%, 0.270 |  |  | 11 | 2252 | 0.03 (-0.05, 0.11) | 47.0%, 0.042 |  |
| **1. quality score** |  |  |  |  | 0.856 |  |  |  |  |  | 0.781 |  |  |  |  |  | 0.815 |
| <9 | 4 | 1589 | 0.03 (-0.01, 0.07) | 0.0%, 0.753 |  |  | 2 | 1621 | 0.01 (-0.03, 0.06) | 0.0%, 0.820 |  |  | 4 | 1648 | -0.01 (-0.05, 0.03) | 0.0%, 0.729 |  |
| >=9 | 19 | 801 | 0.04 (-0.03, 0.10) | 0.0%, 0.949 |  |  | 21 | 915 | 0.00 (-0.05, 0.05) | 21.3%, 0.186 |  |  | 7 | 604 | 0.05 (-0.10, 0.20) | 63.6%, 0.011 |  |
| **2. study design** |  |  |  |  | 0.985 |  |  |  |  |  | 0.762 |  |  |  |  |  | 0.455 |
| RCT | 4 | 1890 | 0.03 (-0.01, 0.08) | 0.0%, 0.883 |  |  | 3 | 1667 | 0.01 (-0.03, 0.06) | 0.0%, 0.897 |  |  | 3 | 1638 | -0.02 (-0.06, 0.02) | 0.0%, 0.999 |  |
| Cohort | 19 | 500 | 0.03 (-0.04, 0.10) | 0.0%, 0.931 |  |  | 20 | 869 | 0.00 (-0.05, 0.05) | 24.7%, 0.153 |  |  | 8 | 614 | 0.06 (-0.06, 0.18) | 56.5%, 0.024 |  |
| **3. RA duration** |  |  |  |  | 0.551 |  |  |  |  |  | 0.912 |  |  |  |  |  | 0.402 |
| <6 months | 0 | 0 | - | - |  |  | 2 | 70 | 0.00 (-0.18, 0.19) | 0.0%, 0.624 |  |  | 2 | 136 | -0.04 (-0.17, 0.09) | 0.0%, 0.709 |  |
| >=6 months | 18 | 2311 | 0.04 (-0.00, 0.07) | 0.0%, 0.964 |  |  | 17 | 2404 | 0.01 (-0.03, 0.04) | 33.8%, 0.086 |  |  | 9 | 2116 | 0.05 (-0.05, 0.16) | 56.3%, 0.019 |  |
| unclear | 5 | 79 | -0.01 (-0.17, 0.14) | 0.0%, 0.704 |  |  | 4 | 62 | -0.03 (-0.19, 0.13) | 0.0%, 0.818 |  |  | 0 | 0 | - | - |  |
| **4. DAS28** |  |  |  |  | 0.850 |  |  |  |  |  | 0.981 |  |  |  |  |  | 0.988 |
| <=5.1 | 9 | 297 | 0.01 (-0.08, 0.10) | 0.0%, 0.954 |  |  | 7 | 271 | -0.00 (-0.09, 0.09) | 0.0%, 0.855 |  |  | 2 | 91 | 0.07 (-0.09, 0.22) | 0.0%, 0.771 |  |
| >5.1 | 10 | 443 | 0.05 (-0.04, 0.14) | 0.0%, 0.825 |  |  | 14 | 694 | 0.01 (-0.05, 0.06) | 43.2%, 0.043 |  |  | 7 | 564 | 0.03 (-0.11, 0.17) | 61.4%, 0.016 |  |
| unclear | 4 | 1650 | 0.03 (-0.01, 0.08) | 0.0%, 0.460 |  |  | 2 | 1571 | 0.01 (-0.04, 0.05) | 0.0%, 0.940 |  |  | 2 | 1597 | 0.02 (-0.15, 0.20) | 38.4%, 0.203 |  |
| **5. age** |  |  |  |  | 0.636 |  |  |  |  |  | 0.640 |  |  |  |  |  | 0.714 |
| <55 years old | 13 | 366 | 0.06 (-0.02, 0.15) | 0.0%, 0.887 |  |  | 15 | 628 | -0.00 (-0.06, 0.05) | 1.3%, 0.436 |  |  | 7 | 565 | 0.05 (-0.03, 0.13) | 0.0%, 0.656 |  |
| >=55 years old | 9 | 1991 | 0.03 (-0.01, 0.07) | 0.0%, 0.920 |  |  | 8 | 1908 | 0.01 (-0.03, 0.05) | 37.0%, 0.133 |  |  | 4 | 1687 | 0.03 (-0.20, 0.26) | 75.9%, 0.006 |  |
| unclear | 1 | 33 | -0.08 (-0.40, 0.24) | - |  |  | 0 | 0 | - | - |  |  | 0 | 0 | - | - |  |
| **6. drug** |  |  |  |  | 0.795 |  |  |  |  |  | 0.978 |  |  |  |  |  | 0.892 |
| IFX | 9 | 301 | 0.06 (-0.03, 0.16) | 0.0%, 0.661 |  |  | 8 | 270 | 0.02 (-0.09, 0.13) | 54.9%, 0.030 |  |  | 5 | 209 | 0.08 (-0.19, 0.35) | 70.1%, 0.010 |  |
| ADA | 5 | 224 | 0.00 (-0.11, 0.11) | 0.0%, 0.834 |  |  | 4 | 114 | -0.01 (-0.12, 0.11) | 0.0%, 0.801 |  |  | 2 | 136 | -0.04 (-0.17, 0.09) | 0.0%, 0.709 |  |
| ETN | 6 | 1799 | 0.03 (-0.02, 0.07) | 0.0%, 0.959 |  |  | 7 | 1979 | 0.01 (-0.03, 0.05) | 28.6%, 0.210 |  |  | 3 | 1827 | 0.03 (-0.08, 0.15) | 46.9%, 0.152 |  |
| mixed | 3 | 66 | 0.09 (-0.11, 0.28) | 0.0%, 0.530 |  |  | 4 | 173 | -0.01 (-0.08, 0.07) | 0.0%, 0.946 |  |  | 1 | 80 | 0.07 (-0.09, 0.23) | - |  |
| **7. baseline TG** |  |  |  |  | 0.166 |  |  |  |  |  | 0.005 |  |  |  |  |  | 0.084 |
| <1.7 mmol/l | 20 | 2277 | 0.04 (0.00, 0.08) | 0.0%, 0.979 |  |  | 18 | 2377 | 0.02 (-0.01, 0.05) | 0.0%, 0.838 |  |  | 9 | 2161 | 0.05 (-0.03, 0.13) | 45.7%, 0.065 |  |
| >=1.7 mmol/l | 3 | 113 | -0.10 (-0.28, 0.08) | 0.0%, 0.996 |  |  | 5 | 159 | -0.21 (-0.35, -0.07) | 9.5%, 0.352 |  |  | 2 | 91 | -0.21 (-0.41, -0.00) | 0.0%, 0.640 |  |

***** *p*-value for meta regression. Abbreviations: TG, triglyceride; RCT, randomized controlled study; DAS28, 28 joint disease activity score; IFX, Infliximab; ADA, Adalimumab; ETN, Etanercept.

**Supplementary Table 6.** Subgroup analysis of changes in HDL after anti-TNF therapies in short-, mid-, and long-term.

|  | **HDL-short-term** | | | | |  | **HDL-mid-term** | | | | |  | **HDL-long-term** | | | | |
| --- | --- | --- | --- | --- | --- | --- | --- | --- | --- | --- | --- | --- | --- | --- | --- | --- | --- |
|  | **Studies (n)** | **Patients (n)** | **WMD (95% CI)** | **Heterogeneity *I*^2^, *p*-value** | ***p*-value*** |  | **Studies (n)** | **Patients (n)** | **WMD (95% CI)** | **Heterogeneity *I*^2^, *p*-value** | ***p*-value*** |  | **Studies (n)** | **Patients (n)** | **WMD (95% CI)** | **Heterogeneity *I*^2^, *p*-value** | ***p*-value*** |
| **In total** | 27 | 2542 | 0.11 (0.07, 0.15) | 51.8%, 0.001 |  |  | 29 | 2906 | 0.12 (0.06, 0.19) | 84.0%, 0.000 |  |  | 17 | 2695 | 0.11 (0.04, 0.19) | 85.4%, 0.000 |  |
| **1. quality score** |  |  |  |  | 0.263 |  |  |  |  |  | 0.138 |  |  |  |  |  | 0.147 |
| <9 | 4 | 1588 | 0.01 (-0.01, 0.04) | 0.0%, 0.407 |  |  | 4 | 1739 | -0.00 (-0.13, 0.13) | 72.5%, 0.012 |  |  | 7 | 1863 | 0.02 (-0.08, 0.11) | 61.6%, 0.016 |  |
| >=9 | 23 | 954 | 0.12 (0.08, 0.16) | 29.0%, 0.096 |  |  | 25 | 1167 | 0.14 (0.07, 0.22) | 80.1%, 0.000 |  |  | 10 | 832 | 0.16 (0.06, 0.26) | 83.7%, 0.000 |  |
| **2. study design** |  |  |  |  | 0.001 |  |  |  |  |  | 0.869 |  |  |  |  |  | 0.236 |
| RCT | 4 | 1889 | 0.02 (-0.01, 0.04) | 0.0%, 0.562 |  |  | 6 | 1946 | 0.11 (-0.08, 0.30) | 94.9%, 0.000 |  |  | 6 | 1917 | 0.18 (0.01, 0.34) | 94.8%, 0.000 |  |
| Cohort | 23 | 653 | 0.14 (0.10, 0.18) | 8.7%, 0.342 |  |  | 23 | 960 | 0.13 (0.06, 0.19) | 68.3%, 0.000 |  |  | 11 | 778 | 0.06 (-0.00, 0.11) | 27.7%, 0.181 |  |
| **3. RA duration** |  |  |  |  | 0.001 |  |  |  |  |  | 0.012 |  |  |  |  |  | 0.456 |
| <6 months | 0 | 0 | - | - |  |  | 5 | 350 | 0.16 (-0.08, 0.41) | 93.3%, 0.000 |  |  | 5 | 416 | 0.16 (-0.02, 0.34) | 90.8%, 0.000 |  |
| >=6 months | 22 | 2463 | 0.04 (0.02, 0.06) | 0.0%, 0.488 |  |  | 20 | 2494 | 0.03 (0.00, 0.06) | 7.8%, 0.359 |  |  | 12 | 2279 | 0.08 (0.01, 0.15) | 69.4%, 0.000 |  |
| unclear | 5 | 79 | 0.26 (0.14, 0.37) | 46.2%, 0.115 |  |  | 4 | 62 | 0.33 (0.16, 0.49) | 72.0%, 0.013 |  |  | 0 | 0 | - | - |  |
| **4. DAS28** |  |  |  |  | 0.280 |  |  |  |  |  | 0.192 |  |  |  |  |  | 0.297 |
| <=5.1 | 9 | 297 | 0.16 (0.05, 0.28) | 66.5%, 0.002 |  |  | 8 | 286 | 0.23 (0.07, 0.38) | 80.9%, 0.000 |  |  | 3 | 106 | 0.25 (0.01, 0.48) | 71.7%, 0.029 |  |
| >5.1 | 14 | 596 | 0.10 (0.05, 0.14) | 0.0%, 1.000 |  |  | 18 | 1015 | 0.09 (-0.01, 0.18) | 81.6%, 0.000 |  |  | 11 | 958 | 0.07 (-0.04, 0.17) | 83.6%, 0.000 |  |
| unclear | 4 | 1649 | 0.07 (-0.03, 0.17) | 66.0%, 0.032 |  |  | 3 | 1605 | 0.08 (-0.03, 0.19) | 75.0%, 0.018 |  |  | 3 | 1631 | 0.16 (-0.04, 0.35) | 90.6%, 0.000 |  |
| **5. age** |  |  |  |  | 0.449 |  |  |  |  |  | 0.053 |  |  |  |  |  | 0.976 |
| <55 years old | 15 | 426 | 0.14 (0.07, 0.21) | 42.5%, 0.042 |  |  | 20 | 975 | 0.17 (0.07, 0.26) | 84.2%, 0.000 |  |  | 13 | 1009 | 0.12 (0.01, 0.22) | 83.2%, 0.000 |  |
| >=55 years old | 11 | 2083 | 0.08 (0.03, 0.12) | 35.8%, 0.112 |  |  | 9 | 1931 | 0.01 (-0.01, 0.04) | 0.0%, 0.711 |  |  | 4 | 1686 | 0.11 (-0.04, 0.27) | 86.7%, 0.000 |  |
| unclear | 1 | 33 | 0.12 (-0.03, 0.27) | - |  |  | 0 | 0 | - | - |  |  | 0 | 0 | - | - |  |
| **6. drug** |  |  |  |  | 0.394 |  |  |  |  |  | 0.565 |  |  |  |  |  | 0.681 |
| IFX | 12 | 446 | 0.12 (0.07, 0.18) | 12.1%, 0.326 |  |  | 12 | 396 | 0.11 (0.02, 0.19) | 54.6%, 0.012 |  |  | 9 | 408 | 0.15 (0.03, 0.26) | 74.3%, 0.000 |  |
| ADA | 6 | 232 | 0.16 (0.06, 0.26) | 47.0%, 0.093 |  |  | 4 | 114 | 0.22 (0.02, 0.42) | 77.5%, 0.004 |  |  | 2 | 136 | 0.19 (0.02, 0.36) | 63.0%, 0.100 |  |
| ETN | 6 | 1798 | 0.04 (-0.02, 010) | 28.3%, 0.223 |  |  | 9 | 2223 | 0.13 (-0.01, 0.27) | 92.8%, 0.000 |  |  | 5 | 2071 | 0.06 (-0.09, 0.20) | 92.7%, 0.000 |  |
| mixed | 3 | 66 | 0.11 (-0.03, 0.26) | 38.4%, 0.197 |  |  | 4 | 173 | 0.06 (-0.02, 0.14) | 0.0%, 0.450 |  |  | 1 | 80 | 0.01 (-0.22, 0.24) | - |  |
| **7. baseline HDL** |  |  |  |  | 0.149 |  |  |  |  |  | 0.054 |  |  |  |  |  | 0.071 |
| <1.5 mmol/l | 20 | 2080 | 0.13 (0.07, 0.19) | 63.3%, 0.000 |  |  | 18 | 2380 | 0.17 (0.08, 0.26) | 89.4%, 0.000 |  |  | 11 | 2292 | 0.16 (0.07, 0.25) | 89.2%, 0.000 |  |
| >=1.5 mmol/l | 7 | 462 | 0.06 (-0.00, 0.11) | 0.0%, 0.898 |  |  | 11 | 526 | 0.02 (-0.04, 0.08) | 10.6%, 0.343 |  |  | 6 | 403 | -0.01 (-0.14, 0.12) | 56.1%, 0.044 |  |

***** *p*-value for meta regression. Abbreviations: HDL, high-density lipoprotein; RCT, randomized controlled study; DAS28, 28 joint disease activity score; IFX, Infliximab; ADA, Adalimumab; ETN, Etanercept.

**Supplementary Table 7.** Subgroup analysis of changes in LDL after anti-TNF therapies in short-, mid-, and long-term.

|  | **LDL-short-term** | | | | |  | **LDL-mid-term** | | | | |  | **LDL-long-term** | | | | |
| --- | --- | --- | --- | --- | --- | --- | --- | --- | --- | --- | --- | --- | --- | --- | --- | --- | --- |
|  | **Studies (n)** | **Patients (n)** | **WMD (95% CI)** | **Heterogeneity *I*^2^, *p*-value** | ***p*-value*** |  | **Studies (n)** | **Patients (n)** | **WMD (95% CI)** | **Heterogeneity *I*^2^, *p*-value** | ***p*-value*** |  | **Studies (n)** | **Patients (n)** | **WMD (95% CI)** | **Heterogeneity *I*^2^, *p*-value** | ***p*-value*** |
| **In total** | 18 | 2253 | 0.06 (0.01, 0.12) | 4.3%, 0.404 |  |  | 23 | 2692 | 0.06 (-0.04, 0.15) | 52.1%, 0.002 |  |  | 12 | 2445 | 0.10 (-0.05, 0.24) | 69.2%, 0.000 |  |
| **1. quality score** |  |  |  |  | 0.464 |  |  |  |  |  | 0.416 |  |  |  |  |  | 0.031 |
| <9 | 3 | 1576 | 0.04 (-0.02, 0.11) | 0.0%, 0.924 |  |  | 3 | 1725 | -0.03 (-0.09, 0.03) | 0.0%, 0.986 |  |  | 4 | 1745 | -0.08 (-0.20, 0.04) | 19.3%, 0.294 |  |
| >=9 | 15 | 677 | 0.10 (0.02, 0.18) | 14.4%, 0.292 |  |  | 20 | 967 | 0.08 (-0.04, 0.19) | 51.6%, 0.004 |  |  | 8 | 700 | 0.23 (0.05, 0.41) | 56.7%, 0.024 |  |
| **2. study design** |  |  |  |  | 0.120 |  |  |  |  |  | 0.052 |  |  |  |  |  | 0.691 |
| RCT | 4 | 1889 | 0.04 (-0.02, 0.10) | 0.0%, 0.946 |  |  | 6 | 1947 | 0.23 (-0.05, 0.51) | 86.4%, 0.000 |  |  | 6 | 1918 | 0.14 (-0.13, 0.42) | 81.8%, 0.000 |  |
| Cohort | 14 | 364 | 0.14 (0.04, 0.23) | 11.5%, 0.327 |  |  | 17 | 745 | 0.01 (-0.06, 0.09) | 0.0%, 0.911 |  |  | 6 | 527 | 0.07 (-0.09, 0.22) | 29.0%, 0.218 |  |
| **3. RA duration** |  |  |  |  | 0.114 |  |  |  |  |  | 0.007 |  |  |  |  |  | 0.654 |
| <6 months | 0 | 0 | - | - |  |  | 5 | 350 | 0.31 (-0.05, 0.66) | 76.5%, 0.002 |  |  | 5 | 416 | 0.14 (-0.18, 0.47) | 77.8%, 0.001 |  |
| >=6 months | 14 | 2187 | 0.08 (0.02, 0.13) | 8.8%, 0.356 |  |  | 14 | 2280 | -0.00 (-0.05, 0.05) | 0.0%, 0.784 |  |  | 7 | 2029 | 0.04 (-0.10, 0.18) | 48.5%, 0.070 |  |
| unclear | 4 | 66 | -0.09 (-0.29, 0.11) | 0.0%, 0.780 |  |  | 4 | 62 | -0.11 (-0.33, 0.10) | 0.0%, 0.881 |  |  | 0 | 0 | - | - |  |
| **4. DAS28** |  |  |  |  | 0.198 |  |  |  |  |  | 0.372 |  |  |  |  |  | 0.858 |
| <=5.1 | 7 | 274 | -0.03 (-0.16, 0.10) | 0.0%, 0.935 |  |  | 7 | 271 | -0.03 (-0.16, 0.09) | 0.0%, 0.767 |  |  | 2 | 91 | 0.19 (-0.45, 0.83) | 42.8%, 0.186 |  |
| >5.1 | 8 | 352 | 0.12 (-0.01, 0.24) | 0.0%, 0.937 |  |  | 13 | 815 | 0.11 (-0.06, 0.28) | 63.3%, 0.001 |  |  | 7 | 722 | 0.06 (-0.16, 0.28) | 71.9%, 0.002 |  |
| unclear | 3 | 1627 | 0.07 (0.01, 0.14) | 81.3%, 0.005 |  |  | 3 | 1606 | 0.03 (-0.09, 0.16) | 41.3%, 0.182 |  |  | 3 | 1632 | 0.18 (-0.19, 0.54) | 77.8%, 0.011 |  |
| **5. age** |  |  |  |  | 0.922 |  |  |  |  |  | 0.812 |  |  |  |  |  | 0.529 |
| <55 years old | 9 | 309 | 0.06 (-0.06, 0.18) | 31.1%, 0.170 |  |  | 16 | 864 | 0.06 (-0.10, 0.21) | 62.5%, 0.000 |  |  | 9 | 838 | 0.13 (-0.06, 0.32) | 67.8%, 0.002 |  |
| >=55 years old | 8 | 1911 | 0.06 (0.01, 0.12) | 0.0%, 0.527 |  |  | 7 | 1828 | -0.00 (-0.06, 0.05) | 0.0%, 0.796 |  |  | 3 | 1607 | -0.05 (-0.18, 0.09) | 11.0%, 0.325 |  |
| unclear | 1 | 33 | 0.11 (-0.35, 0.57) | - |  |  | 0 | 0 | - | - |  |  | 0 | 0 | - | - |  |
| **6. drug** |  |  |  |  | 0.148 |  |  |  |  |  | 0.949 |  |  |  |  |  | 0.803 |
| IFX | 6 | 201 | 0.20 (0.04, 0.36) | 36.6%, 0.163 |  |  | 7 | 215 | 0.05 (-0.11, 0.21) | 2.6%, 0.405 |  |  | 4 | 157 | 0.24 (-0.11, 0.59) | 52.3%, 0.098 |  |
| ADA | 5 | 223 | 0.08 (-0.09, 0.24) | 0.0%, 0.856 |  |  | 9 | 2224 | -0.02 (-0.25, 0.21) | 0.0%, 0.869 |  |  | 2 | 136 | 0.18 (-0.04, 0.39) | 0.0%, 0.459 |  |
| ETN | 6 | 1799 | 0.03 (-0.03, 0.09) | 0.0%, 0.809 |  |  | 3 | 139 | 0.08 (-0.10, 0.26) | 78.8%, 0.000 |  |  | 5 | 2072 | 0.03 (-0.20, 0.26) | 82.1%, 0.000 |  |
| mixed | 1 | 30 | 0.20 (0.02, 0.38) | - |  |  | 4 | 114 | 0.05 (-0.09, 0.18) | 0.0%, 0.614 |  |  | 1 | 80 | 0.00 (-0.30, 0.30) | - |  |
| **7. baseline LDL** |  |  |  |  | 0.062 |  |  |  |  |  | 0.430 |  |  |  |  |  | 0.067 |
| <3.4 mmol/l | 14 | 2101 | 0.04 (-0.01, 0.10) | 0.4%, 0.444 |  |  | 15 | 2345 | 0.08 (-0.05, 0.22) | 67.7%, 0.000 |  |  | 10 | 2286 | 0.16 (0.00, 0.32) | 70.4%, 0.000 |  |
| >=3.4 mmol/l | 4 | 152 | 0.20 (0.06, 0.33) | 0.0%, 0.874 |  |  | 8 | 347 | 0.02 (-0.09, 0.14) | 0.0%, 0.919 |  |  | 2 | 159 | -0.25 (-0.53, 0.02) | 14.5%, 0.280 |  |

***** *p*-value for meta regression. Abbreviations: LDL, low-density lipoprotein; RCT, randomized controlled study; DAS28, 28 joint disease activity score; IFX, Infliximab; ADA, Adalimumab; ETN, Etanercept.

**Supplementary Table 8.** Subgroup analysis of changes in AI after anti-TNF therapies in short-, mid-, and long-term.

|  | **AI-short-term** | | | | |  | **AI-mid-term** | | | | |  | **AI-long-term** | | | |
| --- | --- | --- | --- | --- | --- | --- | --- | --- | --- | --- | --- | --- | --- | --- | --- | --- |
|  | **Studies (n)** | **Patients (n)** | **WMD (95% CI)** | **Heterogeneity *I*^2^, *p*-value** | ***p*-value*** |  | **Studies (n)** | **Patients (n)** | **WMD (95% CI)** | **Heterogeneity *I*^2^, *p*-value** | ***p*-value*** |  | **Studies (n)** | **Patients (n)** | **WMD (95% CI)** | **Heterogeneity *I*^2^, *p*-value** |
| **In total** | 12 | 515 | -0.12 (-0.35, 0.10) | 54.3%, 0.012 |  |  | 16 | 766 | -0.12 (-0.29, 0.06) | 64.4%, 0.000 |  |  | 7 | 594 | 0.03 (-0.11, 0.17) | 19.1%, 0.284 |
| **1. quality score** |  |  |  |  | - |  |  |  |  |  | 0.418 |  |  |  |  |  |
| <9 | 0 | 0 | - | - |  |  | 1 | 80 | 0.20 (-0.10, 0.50) | - |  |  | 1 | 80 | 0.10 (-0.21, 0.41) | - |
| >=9 | 12 | 515 | -0.12 (-0.35, 0.10) | 54.3%, 0.012 |  |  | 15 | 686 | -0.15 (-0.34, 0.04) | 64.0%, 0.000 |  |  | 6 | 514 | 0.01 (-0.15, 0.17) | 30.3%, 0.208 |
| **2. study design** |  |  |  |  | 0.102 |  |  |  |  |  | 0.852 |  |  |  |  |  |
| RCT | 1 | 145 | -0.01 (-0.24, 0.22) | - |  |  | 2 | 55 | -0.22 (-0.97, 0.52) | 62.3%, 0.103 |  |  | 1 | 35 | -0.89 (-1.72, -0.06) | - |
| Cohort | 11 | 370 | -0.15 (-0.42, 0.12) | 57.7%, 0.009 |  |  | 14 | 711 | -0.11 (-0.30, 0.08) | 67.0%, 0.000 |  |  | 6 | 559 | 0.06 (-0.09, 0.20) | 0.0%, 0.769 |
| **3. RA duration** |  |  |  |  | 0.004 |  |  |  |  |  | 0.011 |  |  |  |  |  |
| <6 months | 0 | 0 | - | - |  |  | 3 | 105 | -0.17 (-0.50, 0.17) | 25.9%, 0.259 |  |  | 2 | 85 | -0.26 (-0.59, 0.07) | 62.2%, 0.104 |
| >=6 months | 8 | 449 | 0.02 (-0.13, 0.16) | 0.0%, 0.678 |  |  | 9 | 599 | 0.05 (-0.08, 0.17) | 19.1%, 0.273 |  |  | 5 | 509 | 0.10 (-0.06, 0.26) | 0.0%, 0.888 |
| unclear | 4 | 66 | -0.73 (-1.27, -0.19) | 41.7%, 0.161 |  |  | 4 | 62 | -0.82 (-1.45, -0.18) | 75.3%, 0.007 |  |  | 0 | 0 | - | - |
| **4. DAS28** |  |  |  |  | 0.005 |  |  |  |  |  | 0.040 |  |  |  |  |  |
| <=5.1 | 3 | 33 | -0.94 (-1.36, -0.52) | 0.0%, 0.742 |  |  | 4 | 113 | -0.76 (-1.61, 0.09) | 88.7%, 0.000 |  |  | 1 | 80 | 0.10 (-0.21, 0.41) | - |
| >5.1 | 8 | 426 | 0.00 (-0.15, 0.15) | 0.0%, 0.863 |  |  | 11 | 618 | -0.01 (-0.12, 0.09) | 0.0%, 0.487 |  |  | 4 | 423 | 0.04 (-0.13, 0.21) | 0.0%, 0.491 |
| unclear | 1 | 56 | -0.12 (-0.35, 0.10) | - |  |  | 1 | 35 | -0.67 (-1.45, 0.11) | - |  |  | 2 | 91 | -0.17 (-0.60, 0.26) | 74.8%, 0.046 |
| **5. age** |  |  |  |  | 0.581 |  |  |  |  |  | 0.168 |  |  |  |  |  |
| <55 years old | 8 | 313 | -0.23 (-0.57, 0.11) | 68.8%, 0.002 |  |  | 13 | 637 | -0.20 (-0.40, 0.00) | 66.4%, 0.000 |  |  | 6 | 539 | 0.02 (-0.13, 0.17) | 29.2%, 0.216 |
| >=55 years old | 3 | 169 | -0.01 (-0.25, 0.24) | 0.0%, 0.838 |  |  | 3 | 129 | 0.21 (-0.06, 0.47) | 0.0%, 0.403 |  |  | 1 | 55 | 0.17 (-0.31, 0.65) | - |
| unclear | 1 | 33 | 0.48 (-0.75, 1.71) | - |  |  | 0 | 0 | - | - |  |  | 0 | 0 | - | - |
| **6. drug** |  |  |  |  | 0.475 |  |  |  |  |  | 0.573 |  |  |  |  |  |
| IFX | 7 | 307 | -0.02 (-0.32, 0.28) | 55.7%, 0.035 |  |  | 7 | 237 | -0.03 (-0.41, 0.35) | 68.8%, 0.004 |  |  | 4 | 198 | 0.08 (-0.20, 0.35) | 52.0%, 0.100 |
| ADA | 4 | 196 | -0.22 (-0.67, 0.22) | 59.3%, 0.061 |  |  | 3 | 92 | -0.37 (-0.85, 0.12) | 70.7%, 0.033 |  |  | 1 | 50 | -0.14 (-0.50, 0.22) | - |
| ETN | 1 | 12 | -0.70 (-1.45, 0.05) | - |  |  | 3 | 294 | -0.39 (-1.02, 0.23) | 78.4%, 0.010 |  |  | 1 | 266 | 0.03 (-0.21, 0.27) | - |
| mixed | 0 | 0 | - | - |  |  | 3 | 143 | -0.00 (-0.20, 0.19) | 32.3%, 0.228 |  |  | 1 | 80 | 0.10 (-0.21, 0.41) | - |

***** *p*-value for meta regression. Abbreviations: AI, atherogenic index; RCT, randomized controlled study; DAS28, 28 joint disease activity score; IFX, Infliximab; ADA, Adalimumab; ETN, Etanercept.

**Supplementary Table 9.** Subgroup analysis of changes in Apo A1 after anti-TNF therapies in short-, mid-, and long-term.

|  | **Apo A1-short-term** | | | |  | **Apo A1-mid-term** | | | |  | **Apo A1-long-term** | | | |
| --- | --- | --- | --- | --- | --- | --- | --- | --- | --- | --- | --- | --- | --- | --- |
|  | **Studies (n)** | **Patients (n)** | **WMD (95% CI)** | **Heterogeneity *I*^2^, *p*-value** |  | **Studies (n)** | **Patients (n)** | **WMD (95% CI)** | **Heterogeneity *I*^2^, *p*-value** |  | **Studies (n)** | **Patients (n)** | **WMD (95% CI)** | **Heterogeneity *I*^2^, *p*-value** |
| **In total** | 11 | 600 | 0.07 (0.04, 0.10) | 0.0%, 0.590 |  | 11 | 869 | 0.03 (-0.00, 0.06) | 0.0%, 0.981 |  | 7 | 581 | 0.03 (-0.01, 0.07) | 25.5%, 0.234 |
| **1. quality score** |  |  |  |  |  |  |  |  |  |  |  |  |  |  |
| <9 | 0 | 0 | - | - |  | 0 | 0 | - | - |  | 0 | 0 | - | - |
| >=9 | 11 | 600 | 0.07 (0.04, 0.10) | 0.0%, 0.590 |  | 11 | 869 | 0.03 (-0.00, 0.06) | 0.0%, 0.981 |  | 7 | 581 | 0.03 (-0.01, 0.07) | 25.5%, 0.234 |
| **2. study design** |  |  |  |  |  |  |  |  |  |  |  |  |  |  |
| RCT | 2 | 204 | 0.03 (-0.03, 0.09) | 0.0%, 0.756 |  | 2 | 202 | 0.02 (-0.05, 0.09) | 0.0%, 1.000 |  | 1 | 18 | 0.19 (0.02, 0.36) | - |
| Cohort | 9 | 396 | 0.09 (0.05, 0.12) | 0.0%, 0.631 |  | 9 | 667 | 0.03 (-0.00, 0.06) | 0.0%, 0.939 |  | 6 | 563 | 0.02 (-0.02, 0.06) | 0.0%, 0.462 |
| **3. RA duration** |  |  |  |  |  |  |  |  |  |  |  |  |  |  |
| <6 months | 0 | 0 | - | - |  | 0 | 0 | - | - |  | 1 | 18 | 0.19 (0.02, 0.36) | - |
| >=6 months | 11 | 600 | 0.07 (0.04, 0.10) | 0.0%, 0.590 |  | 9 | 744 | 0.03 (-0.00, 0.06) | 0.0%, 0.953 |  | 6 | 563 | 0.02 (-0.02, 0.06) | 0.0%, 0.462 |
| unclear | 0 | 0 | - | - |  | 2 | 125 | 0.04 (-0.05, 0.12) | 0.0%, 0.587 |  | 0 | 0 | - | - |
| **4. DAS28** |  |  |  |  |  |  |  |  |  |  |  |  |  |  |
| <=5.1 | 3 | 214 | 0.04 (-0.02, 0.09) | 0.0%, 0.865 |  | 2 | 202 | 0.02 (-0.05, 0.09) | 0.0%, 1.000 |  | 1 | 18 | 0.19 (0.02, 0.36) | - |
| >5.1 | 7 | 356 | 0.08 (0.03, 0.12) | 0.0%, 0.522 |  | 8 | 637 | 0.03 (-0.01, 0.06) | 0.0%, 0.938 |  | 6 | 563 | 0.02 (-0.02, 0.06) | 0.0%, 0.462 |
| unclear | 1 | 30 | 0.12 (0.04, 0.20) | - |  | 1 | 30 | 0.06 (-0.02, 0.14) | - |  | 0 | 0 | - | - |
| **5. age** |  |  |  |  |  |  |  |  |  |  |  |  |  |  |
| <55 years old | 1 | 10 | 0.06 (-0.05, 0.17) | - |  | 1 | 266 | 0.03 (-0.03, 0.09) | - |  | 1 | 266 | 0.00 (-0.07, 0.07) | - |
| >=55 years old | 10 | 590 | 0.07 (0.04, 0.11) | 0.0%, 0.499 |  | 10 | 603 | 0.03 (-0.01, 0.06) | 0.0%, 0.964 |  | 6 | 315 | 0.05 (-0.00, 0.10) | 24.7%, 0.249 |
| unclear | 0 | 0 | - | - |  | 0 | 0 | - | - |  | 0 | 0 | - | - |
| **6. drug** |  |  |  |  |  |  |  |  |  |  |  |  |  |  |
| IFX | 5 | 250 | 0.09 (0.03, 0.14) | 0.0%, 0.885 |  | 4 | 240 | 0.01 (-0.04, 0.07) | 0.0%, 0.611 |  | 4 | 213 | 0.04 (-0.02, 0.10) | 40.9%, 0.166 |
| ADA | 2 | 56 | 0.12 (0.02, 0.22) | 0.0%, 0.572 |  | 1 | 42 | 0.02 (-0.12, 0.16) | - |  | 1 | 42 | 0.01 (-0.12, 0.14) | - |
| ETN | 3 | 264 | 0.02 (-0.04, 0.07) | 0.0%, 0.703 |  | 3 | 432 | 0.03 (-0.02, 0.07) | 0.0%, 0.985 |  | 2 | 326 | 0.03 (-0.03, 0.09) | 64.2%, 0.095 |
| mixed | 1 | 30 | 0.12 (0.04, 0.20) | - |  | 3 | 155 | 0.05 (-0.01, 0.11) | 0.0%, 0.801 |  | 0 | 0 | - | - |

Abbreviations: Apo A1, apolipoprotein A1; RCT, randomized controlled study; DAS28, 28 joint disease activity score; IFX, Infliximab; ADA, Adalimumab; ETN, Etanercept.

**Supplementary Table 10.** Subgroup analysis of changes in Apo B after anti-TNF therapies in short-, mid-, and long-term.

|  | **Apo B-short-term** | | | |  | **Apo B-mid-term** | | | |  | **Apo B-long-term** | | | |
| --- | --- | --- | --- | --- | --- | --- | --- | --- | --- | --- | --- | --- | --- | --- |
|  | **Studies (n)** | **Patients (n)** | **WMD (95% CI)** | **Heterogeneity *I*^2^, *p*-value** |  | **Studies (n)** | **Patients (n)** | **WMD (95% CI)** | **Heterogeneity *I*^2^, *p*-value** |  | **Studies (n)** | **Patients (n)** | **WMD (95% CI)** | **Heterogeneity *I*^2^, *p*-value** |
| **In total** | 10 | 544 | 0.02 (-0.01, 0.05) | 0.0%, 0.651 |  | 10 | 752 | 0.00 (-0.02, 0.02) | 0.0%, 0.720 |  | 8 | 616 | 0.01 (-0.02, 0.03) | 0.0%, 0.877 |
| **1. quality score** |  |  |  |  |  |  |  |  |  |  |  |  |  |  |
| <9 | 0 | 0 | - | - |  | 0 | 0 | - | - |  | 0 | 0 | - | - |
| >=9 | 10 | 544 | 0.02 (-0.01, 0.05) | 0.0%, 0.651 |  | 10 | 752 | 0.00 (-0.02, 0.02) | 0.0%, 0.720 |  | 8 | 616 | 0.01 (-0.02, 0.03) | 0.0%, 0.877 |
| **2. study design** |  |  |  |  |  |  |  |  |  |  |  |  |  |  |
| RCT | 2 | 204 | 0.00 (-0.04, 0.05) | 0.0%, 0.531 |  | 2 | 141 | 0.02 (-0.03, 0.07) | 0.0%, 0.606 |  | 2 | 53 | 0.01 (-0.08, 0.10) | 0.0%, 0.354 |
| Cohort | 8 | 340 | 0.03 (-0.01, 0.07) | 0.0%, 0.569 |  | 8 | 611 | -0.00 (-0.03, 0.02) | 0.0%, 0.613 |  | 6 | 563 | 0.01 (-0.02, 0.03) | 0.0%, 0.817 |
| **3. RA duration** |  |  |  |  |  |  |  |  |  |  |  |  |  |  |
| <6 months | 0 | 0 | - | - |  | 1 | 35 | 0.00 (-0.09, 0.09) | - |  | 2 | 53 | 0.01 (-0.08, 0.10) | 0.0%, 0.354 |
| >=6 months | 10 | 544 | 0.02 (-0.01, 0.05) | 0.0%, 0.651 |  | 8 | 688 | 0.00 (-0.02, 0.03) | 0.0%, 0.518 |  | 6 | 563 | 0.01 (-0.02, 0.03) | 0.0%, 0.817 |
| unclear | 0 | 0 | - | - |  | 1 | 29 | 0.00 (-0.15, 0.15) | - |  | 0 | 0 | - | - |
| **4. DAS28** |  |  |  |  |  |  |  |  |  |  |  |  |  |  |
| <=5.1 | 3 | 214 | 0.00 (-0.04, 0.05) | 0.0%, 0.732 |  | 1 | 106 | 0.03 (-0.03, 0.09) | - |  | 1 | 18 | 0.07 (-0.09, 0.23) | - |
| >5.1 | 7 | 330 | 0.03 (-0.01, 0.07) | 0.0%, 0.493 |  | 8 | 611 | -0.00 (-0.03, 0.02) | 0.0%, 0.613 |  | 6 | 563 | 0.01 (-0.02, 0.03) | 0.0%, 0.817 |
| unclear | 0 | 0 | - | - |  | 1 | 35 | 0.00 (-0.09, 0.09) | - |  | 1 | 35 | -0.02 (-0.13, 0.09) | - |
| **5. age** |  |  |  |  |  |  |  |  |  |  |  |  |  |  |
| <55 years old | 2 | 29 | 0.09 (-0.05, 0.23) | 0.0%, 0.373 |  | 3 | 320 | -0.00 (-0.03, 0.03) | 52.3%, 0.123 |  | 2 | 301 | 0.01 (-0.03, 0.04) | 0.0%, 0.604 |
| >=55 years old | 8 | 515 | 0.02 (-0.01, 0.05) | 0.0%, 0.651 |  | 7 | 432 | 0.01 (-0.03, 0.04) | 0.0%, 0.934 |  | 6 | 315 | 0.01 (-0.03, 0.04) | 0.0%, 0.728 |
| unclear | 0 | 0 | - | - |  | 0 | 0 | - | - |  | 0 | 0 | - | - |
| **6. drug** |  |  |  |  |  |  |  |  |  |  |  |  |  |  |
| IFX | 5 | 224 | 0.04 (-0.00, 0.09) | 0.0%, 0.800 |  | 5 | 249 | 0.02 (-0.02, 0.06) | 0.0%, 0.475 |  | 5 | 248 | 0.00 (-0.04, 0.04) | 0.0%, 0.915 |
| ADA | 2 | 56 | 0.08 (-0.03, 0.18) | 0.0%, 0.868 |  | 1 | 42 | -0.06 (-0.19, 0.07) | - |  | 1 | 42 | -0.07 (-0.20, 0.06) | - |
| ETN | 3 | 264 | -0.01 (-0.05, 0.03) | 0.0%, 0.533 |  | 3 | 432 | -0.00 (-0.03, 0.03) | 0.0%, 0.553 |  | 2 | 326 | 0.02 (-0.02, 0.05) | 0.0%, 0.502 |
| mixed | 0 | 0 | - | - |  | 1 | 29 | 0.00 (-0.15, 0.15) | - |  | 0 | 0 | - | - |

Abbreviations: Apo B, apolipoprotein B; RCT, randomized controlled study; DAS28, 28 joint disease activity score; IFX, Infliximab; ADA, Adalimumab; ETN, Etanercept.

**Supplementary Table 11.** Subgroup analysis of changes in Apo B/Apo A1 after anti-TNF therapies in short-, mid-, and long-term.

|  | **Apo B/Apo A1-short-term** | | | |  | **Apo B/Apo A1-mid-term** | | | |  | **Apo B/Apo A1-long-term** | | | |
| --- | --- | --- | --- | --- | --- | --- | --- | --- | --- | --- | --- | --- | --- | --- |
|  | **Studies (n)** | **Patients (n)** | **WMD (95% CI)** | **Heterogeneity *I*^2^, *p*-value** |  | **Studies (n)** | **Patients (n)** | **WMD (95% CI)** | **Heterogeneity *I*^2^, *p*-value** |  | **Studies (n)** | **Patients (n)** | **WMD (95% CI)** | **Heterogeneity *I*^2^, *p*-value** |
| **In total** | 5 | 256 | -0.02 (-0.05, 0.02) | 0.0%, 0.825 |  | 7 | 587 | -0.02 (-0.05, 0.00) | 0.0%, 0.940 |  | 7 | 576 | -0.02 (-0.04, 0.01) | 0.0%, 0.789 |
| **1. quality score** |  |  |  |  |  |  |  |  |  |  |  |  |  |  |
| <9 | 0 | 0 | - | - |  | 0 | 0 | - | - |  | 0 | 0 | - | - |
| >=9 | 5 | 256 | -0.02 (-0.05, 0.02) | 0.0%, 0.825 |  | 7 | 587 | -0.02 (-0.05, 0.00) | 0.0%, 0.940 |  | 7 | 576 | -0.02 (-0.04, 0.01) | 0.0%, 0.789 |
| **2. study design** |  |  |  |  |  |  |  |  |  |  |  |  |  |  |
| RCT | 0 | 0 | - | - |  | 0 | 0 | - | - |  | 1 | 18 | -0.01 (-0.12, 0.10) | - |
| Cohort | 5 | 256 | -0.02 (-0.05, 0.02) | 0.0%, 0.825 |  | 7 | 587 | -0.02 (-0.05, 0.00) | 0.0%, 0.940 |  | 6 | 558 | -0.02 (-0.04, 0.01) | 0.0%, 0.678 |
| **3. RA duration** |  |  |  |  |  |  |  |  |  |  |  |  |  |  |
| <6 months | 0 | 0 | - | - |  | 1 | 50 | -0.05 (-0.13, 0.03) | - |  | 2 | 68 | -0.04 (-0.10, 0.03) | 0.0%, 0.575 |
| >=6 months | 5 | 256 | -0.02 (-0.05, 0.02) | 0.0%, 0.825 |  | 5 | 508 | -0.02 (-0.05, 0.01) | 0.0%, 0.866 |  | 5 | 508 | -0.01 (-0.04, 0.01) | 0.0%, 0.658 |
| unclear |  |  |  |  |  | 1 | 29 | -0.02 (-0.14, 0.10) | - |  | 0 | 0 | - | - |
| **4. DAS28** |  |  |  |  |  |  |  |  |  |  |  |  |  |  |
| <=5.1 | 0 | 0 | - | - |  | 0 | 0 | - | - |  | 1 | 18 | -0.01 (-0.12, 0.10) | - |
| >5.1 | 5 | 256 | -0.02 (-0.05, 0.02) | 0.0%, 0.825 |  | 7 | 587 | -0.02 (-0.05, 0.00) | 0.0%, 0.940 |  | 6 | 558 | -0.02 (-0.04, 0.01) | 0.0%, 0.678 |
| unclear | 0 | 0 | - | - |  | 0 | 0 | - | - |  | 0 | 0 | - | - |
| **5. age** |  |  |  |  |  |  |  |  |  |  |  |  |  |  |
| <55 years old | 0 | 0 | - | - |  | 2 | 316 | -0.02 (-0.06, 0.01) | 0.0%, 0.501 |  | 2 | 316 | -0.01 (-0.04, 0.03) | 13.0%, 0.284 |
| >=55 years old | 5 | 256 | -0.02 (-0.05, 0.02) | 0.0%, 0.825 |  | 5 | 271 | -0.02 (-0.06, 0.02) | 0.0%, 0.866 |  | 5 | 260 | -0.02 (-0.06, 0.01) | 0.0%, 0.787 |
| unclear | 0 | 0 | - | - |  | 0 | 0 | - | - |  | 0 | 0 | - | - |
| **6. drug** |  |  |  |  |  |  |  |  |  |  |  |  |  |  |
| IFX | 2 | 140 | -0.02 (-0.07, 0.03) | 0.0%, 0.338 |  | 2 | 140 | -0.01 (-0.06, 0.03) | 0.0%, 0.343 |  | 3 | 158 | -0.01 (-0.06, 0.03) | 0.0%, 0.610 |
| ADA | 2 | 56 | 0.01 (-0.07, 0.10) | 0.0%, 0.712 |  | 2 | 92 | -0.05 (-0.11, 0.01) | 0.0%, 1.000 |  | 2 | 92 | -0.05 (-0.12, 0.01) | 0.0%, 0.882 |
| ETN | 1 | 60 | -0.02 (-0.10, 0.06) | - |  | 2 | 326 | -0.02 (-0.05, 0.01) | 0.0%, 1.000 |  | 2 | 326 | -0.01 (-0.04, 0.03) | 0.0%, 0.486 |
| mixed | 0 | 0 | - | - |  | 1 | 29 | -0.02 (-0.14, 0.10) | - |  | 0 | 0 | - | - |

Abbreviations: Apo A1, apolipoprotein A1; Apo B, apolipoprotein B; RCT, randomized controlled study; DAS28, 28 joint disease activity score; IFX, Infliximab; ADA, Adalimumab; ETN, Etanercept.

**Supplementary Table 12.** Subgroup analysis of changes in LDL/HDL after anti-TNF therapies in short-, mid-, and long-term.

|  | **LDL/HDL-short-term** | | | |  | **LDL/HDL-mid-term** | | | |  | **LDL/HDL-long-term** | | | |
| --- | --- | --- | --- | --- | --- | --- | --- | --- | --- | --- | --- | --- | --- | --- |
|  | **Studies (n)** | **Patients (n)** | **WMD (95% CI)** | **Heterogeneity *I*^2^, *p*-value** |  | **Studies (n)** | **Patients (n)** | **WMD (95% CI)** | **Heterogeneity *I*^2^, *p*-value** |  | **Studies (n)** | **Patients (n)** | **WMD (95% CI)** | **Heterogeneity *I*^2^, *p*-value** |
| **In total** | 10 | 307 | -0.14 (-0.30, 0.01) | 43.1%, 0.071 |  | 7 | 204 | -0.28 (-0.67, 0.10) | 78.4%, 0.000 |  | 3 | 163 | 0.11 (-0.12, 0.34) | 0.0%, 0.430 |
| **1. quality score** |  |  |  |  |  |  |  |  |  |  |  |  |  |  |
| <9 | 0 | 0 | - | - |  | 0 | 0 | - | - |  | 0 | 0 | - | - |
| >=9 | 10 | 307 | -0.14 (-0.30, 0.01) | 43.1%, 0.071 |  | 7 | 204 | -0.28 (-0.67, 0.10) | 78.4%, 0.000 |  | 3 | 163 | 0.11 (-0.12, 0.34) | 0.0%, 0.430 |
| **2. study design** |  |  |  |  |  |  |  |  |  |  |  |  |  |  |
| RCT | 0 | 0 | - | - |  | 0 | 0 | - | - |  | 0 | 0 | - | - |
| Cohort | 10 | 307 | -0.14 (-0.30, 0.01) | 43.1%, 0.071 |  | 7 | 204 | -0.28 (-0.67, 0.10) | 78.4%, 0.000 |  | 3 | 163 | 0.11 (-0.12, 0.34) | 0.0%, 0.430 |
| **3. RA duration** |  |  |  |  |  |  |  |  |  |  |  |  |  |  |
| <6 months | 0 | 0 | - | - |  | 0 | 0 | - | - |  | 0 | 0 | - | - |
| >=6 months | 6 | 241 | -0.00 (-0.18, 0.18) | 0.0%, 0.980 |  | 4 | 171 | 0.13 (-0.07, 0.33) | 0.0%, 0.936 |  | 3 | 163 | 0.11 (-0.12, 0.34) | 0.0%, 0.430 |
| unclear | 4 | 66 | -0.62 (-0.94, 0.29) | 35.5%, 0.199 |  | 3 | 33 | -0.90 (-1.23, -0.57) | 0.0%, 0.922 |  | 0 | 0 | - | - |
| **4. DAS28** |  |  |  |  |  |  |  |  |  |  |  |  |  |  |
| <=5.1 | 3 | 33 | -0.74 (-1.09, -0.39) | 0.0%, 0.837 |  | 3 | 33 | -0.90 (-1.23, -0.57) | 0.0%, 0.922 |  | 0 | 0 | - | - |
| >5.1 | 6 | 218 | -0.01 (-0.20, 0.19) | 0.0%, 0.959 |  | 4 | 171 | 0.13 (-0.07, 0.33) | 0.0%, 0.936 |  | 2 | 107 | 0.20 (-0.08, 0.47) | 0.0%, 0.545 |
| unclear | 1 | 56 | 0.10 (-0.34, 0.54) | - |  | 0 | 0 | - | - |  | 1 | 56 | -0.10 (-0.53, 0.33) | - |
| **5. age** |  |  |  |  |  |  |  |  |  |  |  |  |  |  |
| <55 years old | 6 | 160 | -0.25 (-0.47, -0.04) | 61.3%, 0.024 |  | 5 | 104 | -0.49 (-1.00, 0.01) | 76.8%, 0.002 |  | 2 | 108 | 0.00 (-0.29, 0.30) | 0.0%, 0.510 |
| >=55 years old | 3 | 114 | -0.04 (-0.28, 0.21) | 0.0%, 0.966 |  | 2 | 100 | 0.15 (-0.10, 0.39) | 0.0%, 0.876 |  | 1 | 55 | 0.27 (-0.09, 0.63) | - |
| unclear | 1 | 33 | 0.35 (-0.62, 1.32) | - |  | 0 | 0 | - | - |  | 0 | 0 | - | - |
| **6. drug** |  |  |  |  |  |  |  |  |  |  |  |  |  |  |
| IFX | 6 | 238 | -0.08 (-0.25, 0.10) | 32.8%, 0.190 |  | 5 | 182 | -0.03 (-0.34, 0.28) | 60.0%, 0.041 |  | 3 | 163 | 0.11 (-0.12, 0.34) | 0.0%, 0.430 |
| ADA | 3 | 57 | -0.39 (-0.90, 0.13) | 56.6%, 0.100 |  | 1 | 10 | -1.02 (-1.71, -0.33) | - |  | 0 | 0 | - | - |
| ETN | 1 | 12 | -0.60 (-1.18, -0.02) | - |  | 1 | 12 | -0.88 (-1.38, -0.38) | - |  | 0 | 0 | - | - |
| mixed | 0 | 0 | - | - |  | 0 | 0 | - | - |  | 0 | 0 | - | - |

Abbreviations: LDL, low-density lipoprotein; HDL, high-density lipoprotein; RCT, randomized controlled study; DAS28, 28 joint disease activity score; IFX, Infliximab; ADA, Adalimumab; ETN, Etanercept.

Reference

1. Higgins JPT, Altman DG, Gøtzsche PC, Jüni P, Moher D, Oxman AD, et al. The Cochrane Collaboration’s tool for assessing risk of bias in randomised trials. BMJ. 2011;343:d5928.

2. Wells G, Shea B, O'Connell D, Peterson J, Welch, Losos M, et al., editors. The Newcastle-Ottawa Scale (NOS) for Assessing the Quality of Nonrandomised Studies in Meta-Analyses2014.

3. Slim K, Nini E, Forestier D, Kwiatkowski F, Panis Y, Chipponi J. Methodological index for non-randomized studies (minors): development and validation of a new instrument. ANZ journal of surgery. 2003;73(9):712-6.

4. Whiting PF, Weswood ME, Rutjes AWS, Reitsma JB, Bossuyt PNM, Kleijnen J. Evaluation of QUADAS, a tool for the quality assessment of diagnostic accuracy studies. BMC Medical Research Methodology. 2006;6(1):9.

5. Wan X, Wang W, Liu J, Tong T. Estimating the sample mean and standard deviation from the sample size, median, range and/or interquartile range. BMC Med Res Methodol. 2014;14(1):135.

6. edited by Julian PTH, Sally G. Cochrane handbook for systematic reviews of interventions: Chichester, West Sussex ; Hoboken NJ : John Wiley &amp; Sons, [2008] ©2008; 2008.

7. Singh JA, Saag KG, Bridges SL, Jr., Akl EA, Bannuru RR, Sullivan MC, et al. 2015 American College of Rheumatology Guideline for the Treatment of Rheumatoid Arthritis. Arthritis care & research. 2016;68(1):1-25.

8. Grundy SM, Stone NJ, Bailey AL, Beam C, Birtcher KK, Blumenthal RS, et al. 2018 AHA/ACC/AACVPR/AAPA/ABC/ACPM/ADA/AGS/APhA/ASPC/NLA/PCNA Guideline on the Management of Blood Cholesterol: A Report of the American College of Cardiology/American Heart Association Task Force on Clinical Practice Guidelines. J Am Coll Cardiol. 2019;73(24):e285-e350.

9. Masic D, Stengaard-Pedersen K, Logstrup BB, Horslev-Petersen K, Hetland ML, Junker P, et al. Similar lipid level changes in early rheumatoid arthritis patients following 1-year treat-to-target strategy with adalimumab plus methotrexate versus placebo plus methotrexate: secondary analyses from the randomised controlled OPERA trial. Rheumatology international. 2021;41(3):543-9.

10. Giles JT, Sattar N, Gabriel S, Ridker PM, Gay S, Warne C, et al. Cardiovascular Safety of Tocilizumab Versus Etanercept in Rheumatoid Arthritis: A Randomized Controlled Trial. Arthritis & rheumatology (Hoboken, NJ). 2020;72(1):31-40.

11. Virone A, Bastard JP, Fellahi S, Capeau J, Rouanet S, Sibilia J, et al. Comparative effect of tumour necrosis factor inhibitors versus other biological agents on cardiovascular risk-associated biomarkers in patients with rheumatoid arthritis. RMD open. 2019;5(2):e000897.

12. O'Neill F, Charakida M, Topham E, McLoughlin E, Patel N, Sutill E, et al. Anti-inflammatory treatment improves high-density lipoprotein function in rheumatoid arthritis. Heart. 2017;103(10):766-73.

13. Gabay C, McInnes IB, Kavanaugh A, Tuckwell K, Klearman M, Pulley J, et al. Comparison of lipid and lipid-associated cardiovascular risk marker changes after treatment with tocilizumab or adalimumab in patients with rheumatoid arthritis. Ann Rheum Dis. 2016;75(10):1806-12.

14. Charles-Schoeman C, Wang X, Lee YY, Shahbazian A, Navarro-Millan I, Yang S, et al. Association of Triple Therapy With Improvement in Cholesterol Profiles Over Two-Year Followup in the Treatment of Early Aggressive Rheumatoid Arthritis Trial. Arthritis & rheumatology (Hoboken, NJ). 2016;68(3):577-86.

15. Bissell LA, Hensor EM, Kozera L, Mackie SL, Burska AN, Nam JL, et al. Improvement in insulin resistance is greater when infliximab is added to methotrexate during intensive treatment of early rheumatoid arthritis-results from the IDEA study. Rheumatology (Oxford, England). 2016;55(12):2181-90.

16. Deodhar A, Bitman B, Yang Y, Collier DH. The effect of etanercept on traditional metabolic risk factors for cardiovascular disease in patients with rheumatoid arthritis. Clin Rheumatol. 2016;35(12):3045-52.

17. Tam LS, Shang Q, Li EK, Lee KL, Leung YY, Ying KY, et al. Infliximab is associated with improvement in arterial stiffness in patients with early rheumatoid arthritisa randomized trial. Arthritis and Rheumatism. 2012;63(10).

18. Kume K, Amano K, Yamada S, Hatta K, Ohta H, Kuwaba N. Tocilizumab monotherapy reduces arterial stiffness as effectively as etanercept or adalimumab monotherapy in rheumatoid arthritis: an open-label randomized controlled trial. The Journal of rheumatology. 2011;38(10):2169-71.

19. Engvall IL, Tengstrand B, Brismar K, Hafstrom I. Infliximab therapy increases body fat mass in early rheumatoid arthritis independently of changes in disease activity and levels of leptin and adiponectin: a randomised study over 21 months. Arthritis research & therapy. 2010;12(5):R197.
